# Targeting fibroblast–endothelial cell interactions in LAM pathogenesis using 3D spheroid models and spatial transcriptomics

**DOI:** 10.1172/jci.insight.187899

**Published:** 2025-02-04

**Authors:** Sinem Koc-Gunel, Emily C. Liu, Lalit K. Gautam, Ben A. Calvert, Shubha Murthy, Noa C. Harriott, Janna C. Nawroth, Beiyun Zhou, Vera P. Krymskaya, Amy L. Ryan

**Affiliations:** 1 Hastings Center for Pulmonary Research; Division of Pulmonary, Critical Care and Sleep Medicine, Department of Medicine; and; 2 Department of Stem Cell Biology and Regenerative Medicine, Keck School of Medicine of University of Southern California, Los Angeles, California, USA.; 3 Department of Internal Medicine II, Infectious Diseases, and; 4 Institute for Medical Virology, University Hospital Frankfurt, Goethe University Frankfurt, Frankfurt am Main, Germany.; 5 Department of Anatomy and Cell Biology, Carver College of Medicine, University of Iowa, Iowa City, Iowa, USA.; 6 Helmholtz Pioneer Campus and Institute of Biological and Medical Imaging, Helmholtz Zentrum München, Neuherberg, Germany.; 7 Division of Pulmonary and Critical Care Medicine, Lung Biology Institute, Perelman School of Medicine, University of Pennsylvania (UPenn), Philadelphia, Pennsylvania, USA.

**Keywords:** Cell biology, Pulmonology, Cell migration/adhesion, Genetic diseases, Protein kinases

## Abstract

Lymphangioleiomyomatosis (LAM) is a progressive lung disease with limited treatments, largely because of an incomplete understanding of its pathogenesis. Lymphatic endothelial cells (LECs) invade LAM cell clusters, which include human melanoma black-45–positive epithelioid cells and smooth muscle α-actin–expressing LAM-associated fibroblasts (LAMFs). Recent evidence shows that LAMFs resemble cancer-associated fibroblasts, with LAMF-LEC interactions contributing to disease progression. To explore these mechanisms, we used spatial transcriptomics on LAM lung tissues and identified a gene cluster enriched in kinase signaling pathways linked to myofibroblasts and coexpressed with LEC markers. Kinase arrays revealed elevated PDGFR and FGFR in LAMFs. Using a 3D coculture spheroid model of primary LAMFs and LECs, we observed increased invasion in LAMF-LEC spheroids compared with non-LAM fibroblasts. Treatment with sorafenib, a multikinase inhibitor, significantly reduced invasion, outperforming rapamycin. We also verified tuberous sclerosis complex 2–deficient renal angiomyolipoma (TSC2-null AML) cells as key VEGF-A secretors; VEGF-A was suppressed by sorafenib in both TSC2-null AML cells and LAMFs. These findings highlight VEGF-A and basic FGF as potential therapeutic targets and suggest multikinase inhibition as a promising strategy for LAM.

## Abstract 2

We used 3D spheroids and spatial transcriptomics to identify LAM-associated fibroblasts and lymphatic endothelial cells as key drivers of LAM, highlighting bFGF and VEGF-A as therapeutic targets.

## Graphical abstract

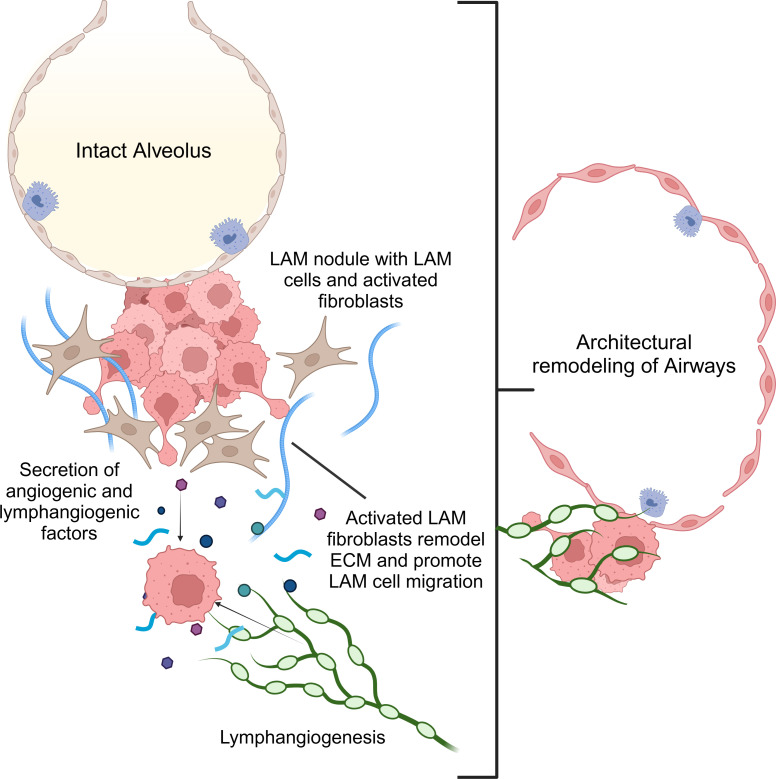

## Introduction

Lymphangioleiomyomatosis (LAM) is a progressive lung disease characterized by the formation of neoplastic lesions, consisting of tumorous smooth muscle-like cells and human melanoma black-45–expressing (HMB-45–expressing) epithelioid-like cells (LAM cells). These lesions lead to cystic destruction of lung tissue and invasion of bronchioles, blood vessels, and lymphatic vessels, ultimately causing severe obstructive lung disease (1, 2). Despite advances in understanding the disease, therapeutic options for LAM remain limited, and it continues to be a debilitating condition primarily affecting women of childbearing age.

One of the major challenges in studying rare lung diseases, like LAM, is the lack of disease-relevant, patient-specific tissues and animal models that accurately reflect the human LAM phenotype (3). The cellular and molecular mechanisms driving the extensive cystic remodeling of pulmonary airspace and parenchyma in LAM are not well understood, resulting in a paucity of known molecular targets for therapeutic intervention. LAM can manifest sporadically or present as part of tuberous sclerosis, driven by somatic or germline mutations in the tuberous sclerosis complex (*TSC1* or *TSC2*) genes (4–10). LAM lesions consist of aggregations of epithelioid-like cells, smooth muscle α-actin–expressing (SMαA-expressing myofibroblast-like cells (1, 3, 11–14), and lymphatic endothelial cells (LECs) (15–21). Yet the impact of these cell types on disease pathogenesis remains poorly understood.

Lung-resident fibroblasts, which have been implicated in other conditions such as interstitial lung disease (ILD), lung cancer, and kidney fibrosis (22–25), are believed to modulate the microenvironment in LAM and are potentially activated in a manner similar to carcinoma-associated fibroblasts. Simultaneously, LECs are recognized as key contributors to the progression of neoplastic lesions, possibly facilitating the trafficking of LAM cells through the lymphatic system, thereby promoting metastasis (26). Several pathways have been suggested in the literature as contributors to LAM cell detachment and migration via the lymphatic system to other organs (17, 27–30).

Understanding the roles of LECs and LAM-associated fibroblasts (LAMFs) is critical for identifying new therapeutic targets and developing more effective treatments. However, the precise contributions of LECs to LAM pathogenesis, including their involvement in lymphangiogenesis and interaction with LAMFs, remain unclear. Most cellular models of LAM study cells in isolation within 2-dimensional (2D) culture environments; these are reductionist and often lose important properties, like differentiation, polarization, cell-cell communication, and interactions with the extracellular matrix (ECM). At the same time, wound healing, inflammatory processes, and hyperproliferation are artificially promoted (31). These limitations hinder our understanding of the intercellular interactions driving LAM pathogenesis, particularly between LAMFs and LECs.

Current therapeutic strategies predominantly target the core *TSC2^–/–^* LAM cells, potentially overlooking the role of simultaneously activated cells, such as LAMFs and LECs, within LAM nodules. These overlooked components may play a crucial role in disease progression, underscoring the need for a more comprehensive approach that addresses interactions between LAM-associated cells and their microenvironment. Rapamycin, an mTOR inhibitor, is the standard therapy for LAM patients with declining lung function (32). It inhibits mTOR complex 1 (mTORC1) activity, preventing the increased cell growth, proliferation, and survival associated with *TSC2* mutations (4, 30, 33–36). Current guidelines recommend rapamycin therapy for patients with a forced expiratory volume (FEV_1_) < 70% compared with the average healthy person (32).

However, LAM shares mechanistic similarities with other neoplastic diseases, including cytokine secretion and the activation of pathways that promote cell growth and migration (37–40). Given this context, investigating multikinase inhibitors that target multiple cell types in the LAM microenvironment is crucial, especially in patients with advanced disease. Sorafenib, a food and drug administration–approved (FDA-approved) multikinase inhibitor, was chosen as a proof-of-concept therapy because of its known effects on tumor cell growth, angiogenesis, and metastasis (41–43). Sorafenib also inhibits eukaryotic translation initiation factor (eIF4E), a factor upregulated in LAM downstream of mTOR activation, and blocks vascular endothelial growth factor receptors 2 and 3 (VEGFR-2 and -3), thereby potentially preventing LAM-associated lymphangiogenesis (3, 43–45). Additionally, sorafenib has shown promise in a mouse model of LAM, improving outcomes alongside rapamycin (46). To better understand the interactions between LAMFs and LECs, we developed 3D spheroid models incorporating these cell types. Our study aimed to elucidate their collaborative roles in disease progression and evaluate the therapeutic potential of sorafenib in modulating these interactions and invasive behaviors.

## Results

### In situ LAM nodule formation verified LEC involvement.

**Figure 1:**
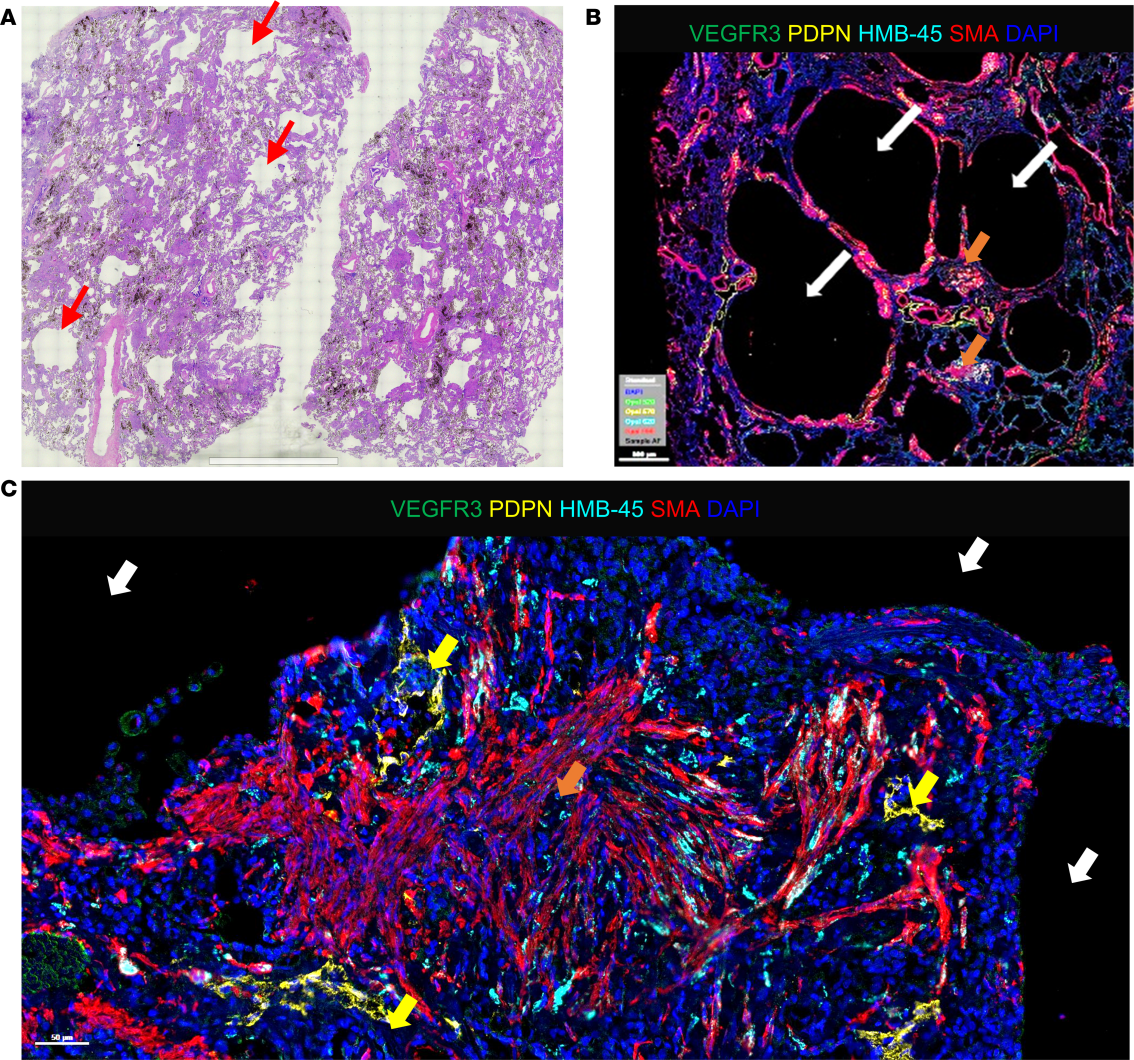
Identification of LAM nodules in patient LAM lung tissue. (**A**) Tiled 20× image of H&E staining of LAM lung tissue; red arrows indicate simplified alveoli. (**B**) Representative images of LAM lung tissues stained with VEGFR3 (green), podoplanin (PDPN) (yellow), HMB-45 (cyan), and SMαA (red). Nuclei are counterstained with DAPI (blue) in all images. White arrows indicate alveolar cysts, orange arrows highlight LAM nodules forming near cysts, and yellow arrows in **C** indicate PDPN and VEGFR3 expressing LEC recruited to LAM nodules. Scale bars represent 2 mm in **A**, 500 μm in **B**, and 50 μm in **C**.

**Figure 2:**
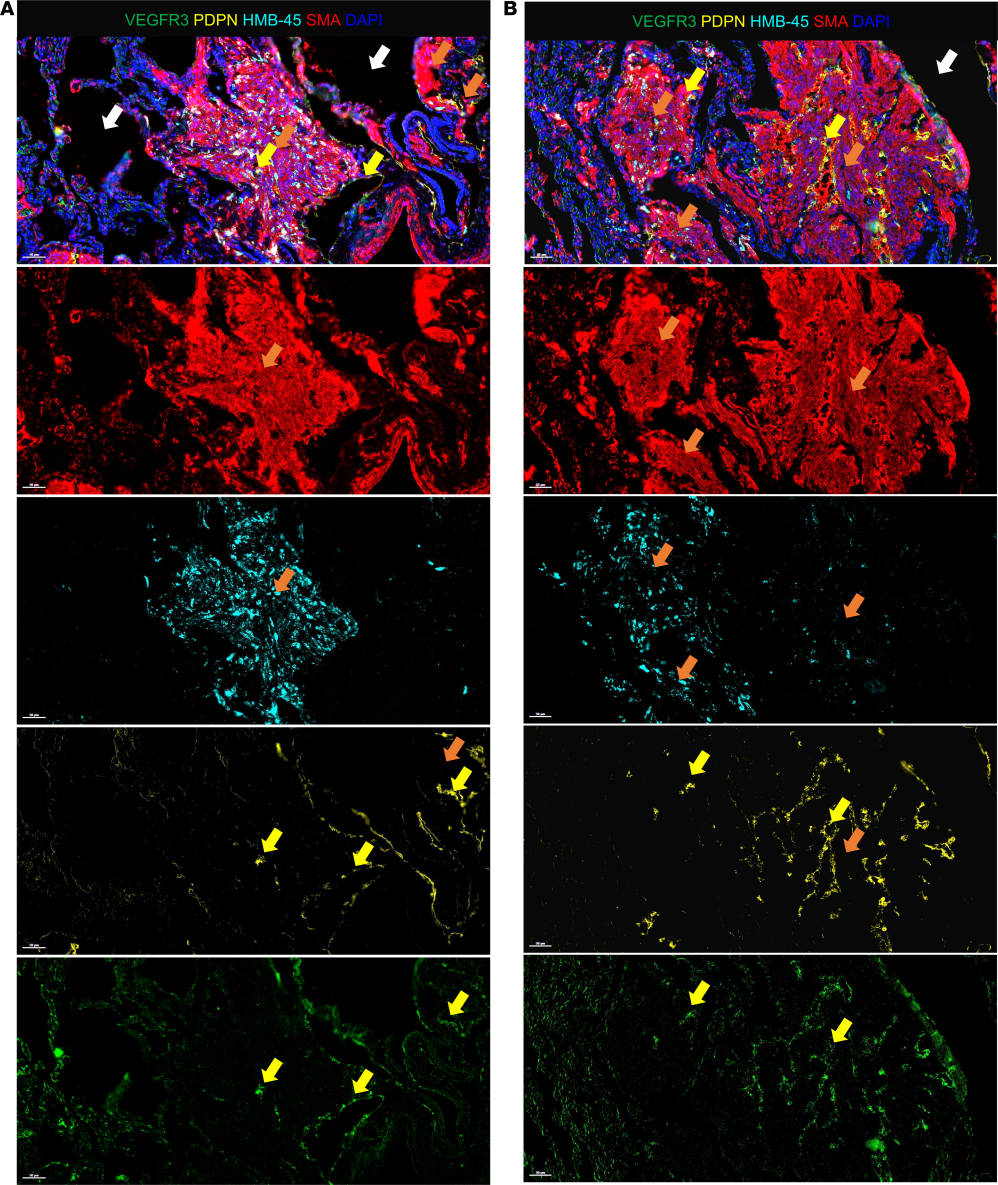
Representative staining of LAM lung tissue highlighting distinct cell populations. (**A** and **B**) Representative images of LAM lung tissues stained with VEGFR3 (green), PDPN (yellow), HMB-45 (cyan), and SMαA (red). Nuclei are counterstained with DAPI (blue) in all images. White arrows indicate alveolar cysts, characteristic structural features of LAM. Orange arrows highlight LAM nodules forming near cysts, with HMB-45 staining serving as the gold standard for diagnosing LAM by identifying melanocytic lineage cells. Yellow arrows indicate LECs expressing both PDPN and VEGFR3, recruited to and integrated within LAM nodules, illustrating the involvement of LECs in LAM pathology.

To validate LEC distribution and association with LAM nodules, we evaluated lung tissues from 2 end-stage LAM donor lungs (Figure 1 and Supplemental Table 1; supplemental material available online with this article; https://doi.org/10.1172/jci.insight.187899DS1). H&E staining of these LAM lung tissues revealed a range of cyst sizes, indicative of alveolar simplification, a hallmark of LAM pathology (Figure 1A, red arrows). Using Opal multiplexed staining, we assessed the colocalization of LAM-associated proteins within a single tissue, providing enhanced spatial insight into cell distribution within the LAM lung. LAM nodules (orange arrows), consisting of SMαA-expressing cells, were observed in close proximity to the cysts (white arrows, Figure 1, B and C; Figure 2; and Supplemental Figure 1). Higher magnification of these regions highlighted the presence of epithelioid-like, HMB-45–positive LAM cells within the SMαA-expressing nodules (Figure 1C and Figure 2, cyan). Additionally, VEGFR3- and PDPN-expressing LECs were clearly identified in and around these HMB-45/SMαA-positive LAM nodules, particularly near cysts, as well as in spindle-shaped, myofibroblast-like LAM cells (Figure 1B, Figure 2, and Supplemental Figure 1). Notably, although PDPN is also expressed in type I pneumocytes, the concurrent expression of PDPN and VEGFR3 is specific to LECs, enabling their distinction from other PDPN-expressing cells. These observations align with the well-documented pathological features of LAM, as described in the literature (2, 12, 20, 47, 48). The presence of LECs invading and surrounding LAM nodules, especially as seen in Figure 2B, further supports their involvement in LAM pathogenesis, consistent with previous studies. The validation of LECs around LAM nodules reinforces their proposed role in the disease (19–21).

### Spatial transcriptomic analysis identifies key LAM core genes within the LAM nodule core.

**Figure 3:**
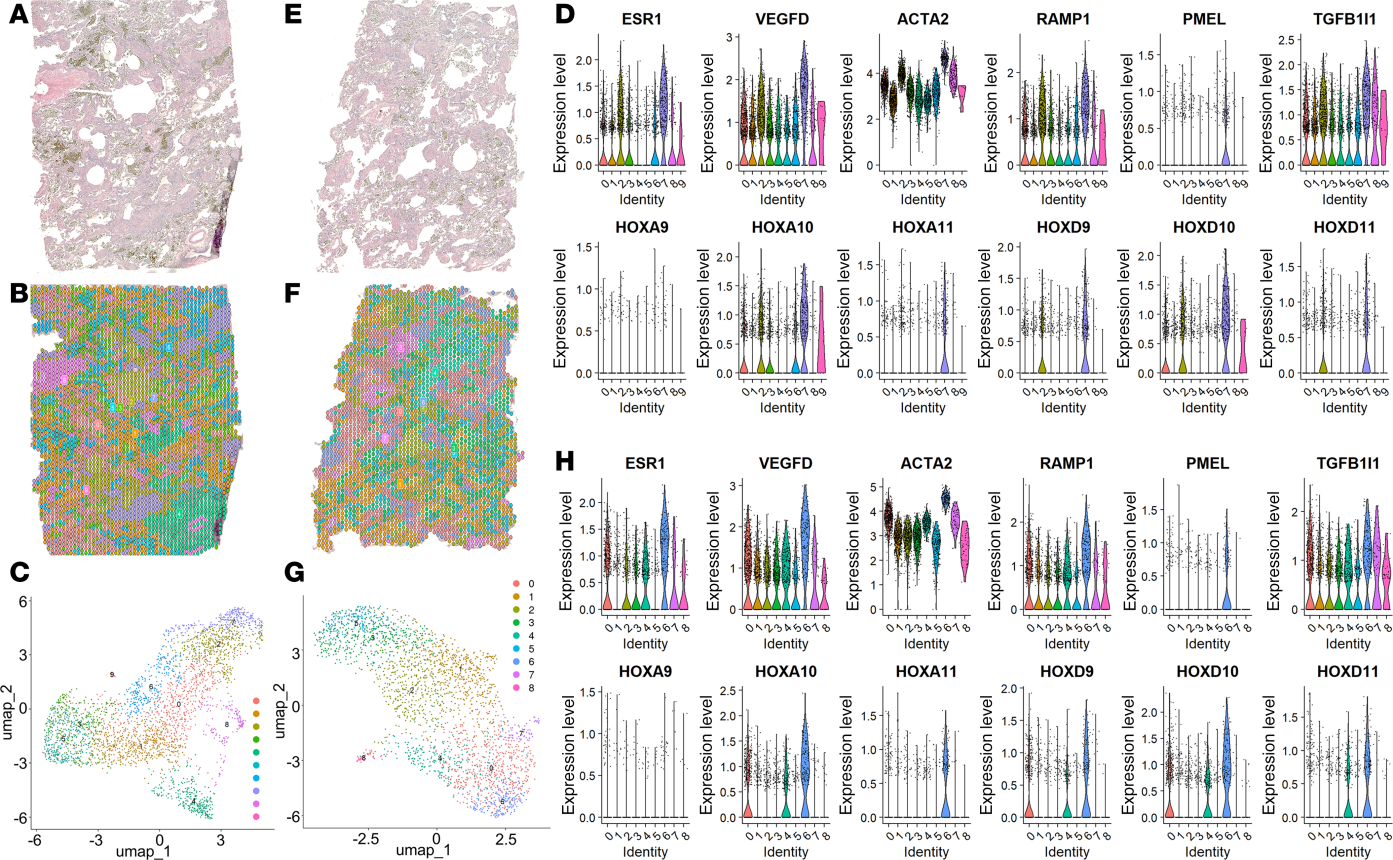
Unbiased clustering of LAM tissue and marker expression. (**A** and **E**) H&E staining of LAM lung tissues used for spatial transcriptomics. (**B** and **F**) Spatial mapping of the gene clusters for each of the lung tissues identified in the uniform manifold approximation and projections (UMAPs) provided in **C** and **G**, where colors represent distinct cellular clusters categorized by gene expression at individual spatial transcriptomic spots, for the combined dataset from both LAM tissues. (**D** and **H**) Violin plots for established LAM-associated genes represented across each cell cluster for LAM_D1 and LAM_D2 lung tissues. TGFB1I1, TGF-β1 interacting protein 1.

**Figure 4:**
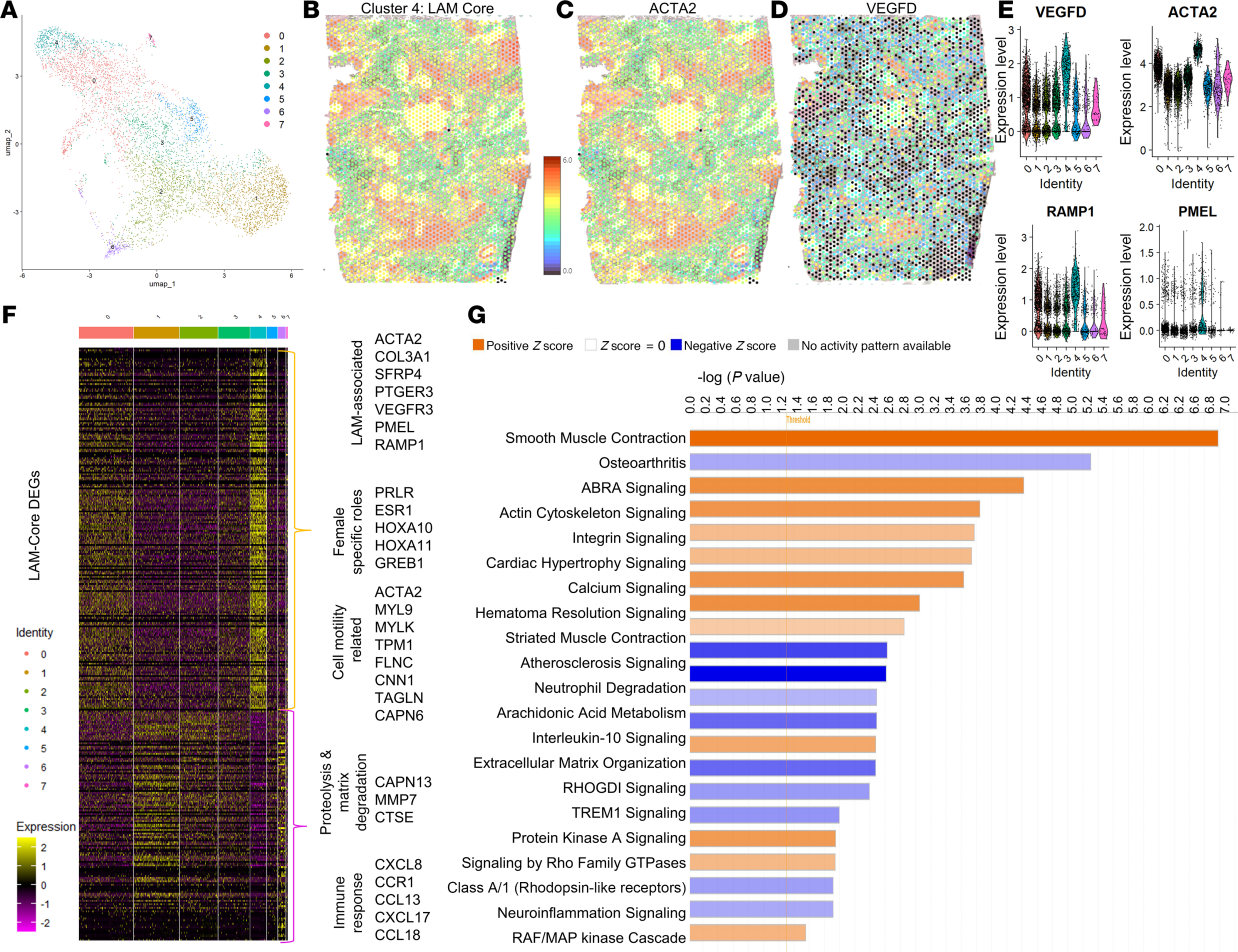
Spatial transcriptomic analysis of LAM tissue highlights LAM-core regions. (**A**) UMAP where colors represent distinct cellular clusters categorized by gene expression at individual spatial transcriptomic spots, for the combined dataset from both LAM tissues. (**B**–**D**) Spatial localization of well-established LAM genes *ACTA2* and *VEGFD* from LAM_D1 lungs showing LAM nodule localization correlating to high gene expression. (**E**) Violin plots of LAM-core genes in the combined dataset identifying cluster 4 as the LAM-core. (**F**) Heatmap for gene expression for the combined dataset where each column represents the color coded cell cluster for all differentially expressed LAM-core–enriched genes with key up- and downregulated genes highlighted. (**G**) Significant IPA canonical pathways’ enrichment in the LAM-core. Orange represents positive *z*-scores, and blue represents negative *z*-scores.

To further investigate the spatial organization of LAM cells, we conducted in situ spatial transcriptomics on 2 independent regions of formalin-fixed, paraffin-embedded (FFPE) LAM donor lung tissue (Figure 3 and Supplemental Figures 2–4). Transcriptomes from 3,343 and 2,595 spots were obtained at a median depth of 8,283 and 7,536 unique molecular identifiers (UMIs)/spot and 4,317 and 4,090 genes/spot for LAM_D1 and LAM_D2, respectively (Supplemental Figure 2). Unbiased clustering and spot feature analysis classified the spots into 10 and 9 clusters for the integrated data of LAM_D1 (Figure 3, A–C) and LAM_D2 (Figure 3, E–G), respectively. Based on both the pathological identification of LAM nodules in the H&E-stained tissue sections (Figure 3, A and E) and the expression of LAM-core genes, such as *ACTA2*, *ESR1*, *PMEL* (*HMB-45*), *RAMP1*, and *VEGFD* (Supplemental Data 1 and 2) (49), which are significantly upregulated in the LAM-core cluster (Figure 3, D and H), we were able to assign clusters 7 and 6 as LAM-core clusters in LAM_D1 and LAM_D2. For subsequent analysis of the transcriptomics data, we integrated both datasets, creating a new UMAP of cell clusters (Figure 4A). Spatial mapping of the turquoise cluster (cluster 4) onto the H&E lung tissue section highlights its association with LAM nodules. We further demonstrate the localization of well-established LAM markers *ACTA2* and *VEGFD* within this cluster (Figure 4, B–D), and the violin plots also include LAM core genes *PMEL* (*HMB-45*) and *RAMP1* (Figure 4E). The heatmap highlights all LAM-core significantly differentially expressed genes (DEGs) between the clusters (Figure 4F). These genes include those well established in the pathogenesis of LAM in addition to *TGFBR3*, Hox family genes (*HOXA9-11* *HOXD9-11I*, *COL14A1*, *COL3A1*, *SFRP4*, *PTGER3*, and *VEGFR3* (Supplemental Data 3). Significantly upregulated genes also include genes that have been directly linked to female physiology and disease, including prolactin receptor (*PRLR*), estrogen receptor 1 (*ESR1*), *HOXA10* and *HOXA11*, and growth regulation by estrogen in breast cancer 1 (*GREB1*), and many genes associated with processes related to muscle contraction, cytoskeletal organization, and cellular motility, including *ACTA2*, myosin light chain 9 (*MYL9*), myosin light chain kinase (*MYLK*), tropomyosin 1 (*TPM1*), filamin C (*FLMC*), transgelin (*TAGLN*), calponin 1 (*CNN1*), and calpain 6 (*CAPN6*). Significantly downregulated genes include genes related to proteolysis and matrix degradation, including matrix metalloproteinase 7 (*MMP7*), cathepsin E (*CTSE*), and calpain 13 (*CAPN13*), which are likely to impact tissue remodeling, and a number of genes related to immune response and inflammation, including *CXCL8* (*IL-8*), *CCR1*, *CCL13*, *CXCL17*, and *CCL18*, which are involved in chemotaxis and immune cell recruitment. To identify significantly enriched canonical pathways in the LAM-core, we conducted Ingenuity Pathway Analysis (IPA) on the LAM-core DEGs, applying a *z*-score threshold of 1 and *P* value of 1.5 (Figure 4G). The most significantly enriched pathways included those related to cell contraction and motility, calcium dynamics, extracellular matrix interactions, and immune cell regulation. Additionally, several kinase signaling pathways were highly represented, including protein kinase A signaling, RAF/MAPK signaling, and Rho-related signaling (Figure 4G; Supplemental Figure 3, C and D; and Supplemental Data 4). The LAM-core significant DEGs were also mapped for their distribution across the sorted cell type annotations based on human single-cell datasets in the Lung Gene Expression Analysis Web Portal (https://research.cchmc.org/pbge/lunggens/mainportal.html) and were found to be predominantly mesenchymal in origin (~65%) with ~15% endothelial, 17% epithelial, and 3% immune cells (Supplemental Figure 3A). The proportion of endothelial related transcripts in the LAM-core is supported by expression of LEC genes *LYVE1*, *PDPN*, and *SOX18* in this same cell cluster (Supplemental Figure 3B).

### Azimuth human lung cell atlas analysis reveals myofibroblasts as key contributors to LAM pathology.

**Figure 5:**
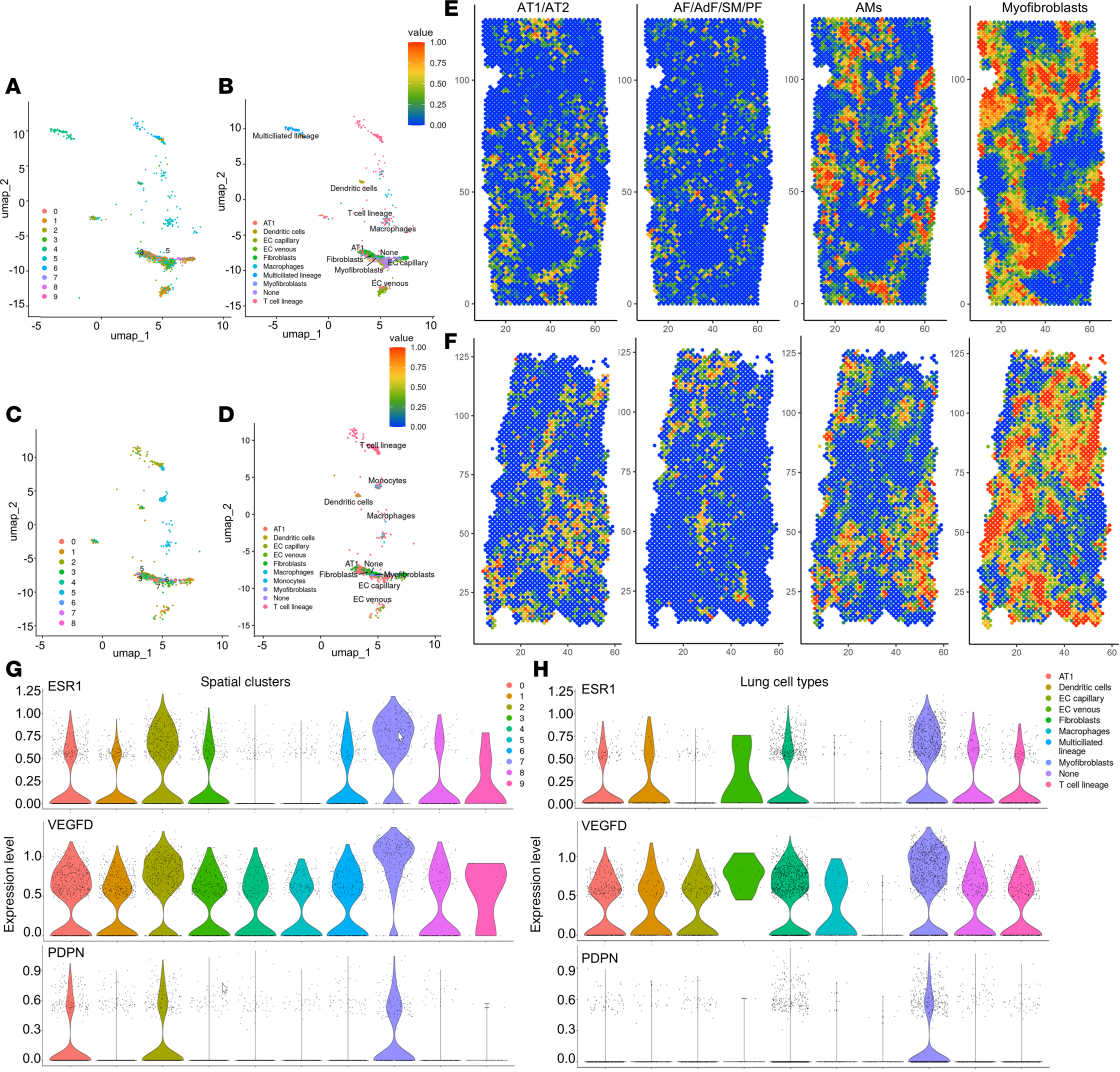
LAM-core–enriched gene signature maps to myofibroblasts in Azimuth: Human Lung v2 (HLCA) database. (**A** and **C**) Spatial transcriptomic gene clusters from LAM_D1 and LAM_D2 tissues, respectively mapped to Lung v2 dataset for level 3 annotation, which refers to the classification of gene expression profiles into more refined cellular subtypes or functional states within the broader lung tissue hierarchy. (**B** and **D**) Spatial gene clusters represented by cell type signatures in human lung tissues for LAM_D1 and LAM_D2, respectively. (**E** and **F**) RCTD images representing LAM_D1 and LAM_D2 cell-associated gene expression. AF, alveolar fibroblasts; AdF, adventitial fibroblasts; AT1, alveolar type 1 cells; SM, smooth muscle cells; PF, peribronchial fibroblasts; AMs, alveolar macrophages. (**G** and **H**) Violin plots for LAM_D1 tissues showing relative gene expression of LAM-core genes and LEC gene *PDPN* with the highest expression in cluster 7 (**G**) mapping to myofibroblasts (**H**).

To assign lung cell types to the UMIs of spatial transcriptomics (spatialomics) data, we mapped our data to a comprehensive expression atlas of the human lung (50, 51). The cell clusters in the spatialomics data sets mapped to 10 lung cell types in the Azimuth atlas (HLCA-Lung v2; https://app.azimuth.hubmapconsortium.org/app/human-lung-v2) (Figure 5, A–D, and Supplemental Table 4). LAM-core genes in cluster 7 (purple, LAM_D1) and cluster 6 (blue, LAM_D2) (Figure 5, A and C, respectively), mapped to myofibroblasts and activated fibroblasts in the LAM lung tissues, after robust cell type decomposition (RCTD) using a single-cell reference-based model (Figure 5, E and F). Mapping the spatial localization of the myofibroblast cluster, we saw that it colocalized with the pathologist-identified LAM nodules, supporting the presence of LAM-core cells in close association with a myofibroblast phenotype (Figure 5, E and F). Looking more closely at the relative expression of LAM-core genes and LEC genes in violin plots, we saw that these genes were most represented in cluster 7 for LAM_D1 (Figure 5G) and in the myofibroblast-mapped population (Figure 5H). To summarize, our spatialomics analysis identified LAM-core genes within a cell cluster that spatially overlaps with pathological LAM nodules in lung tissue. The cells most closely associated with this region include myofibroblasts with endothelial and epithelial cells. The endothelial cells expressed lymphatic endothelial markers, suggesting a close relationship with LECs in the LAM nodules. To further investigate the interactions between LAMFs and LECs, we obtained primary LAM patient–derived fibroblasts and commercially available LECs to establish coculture systems to evaluate cellular interactions.

### LAMFs resemble activated fibroblasts with increased invasion.

**Figure 6:**
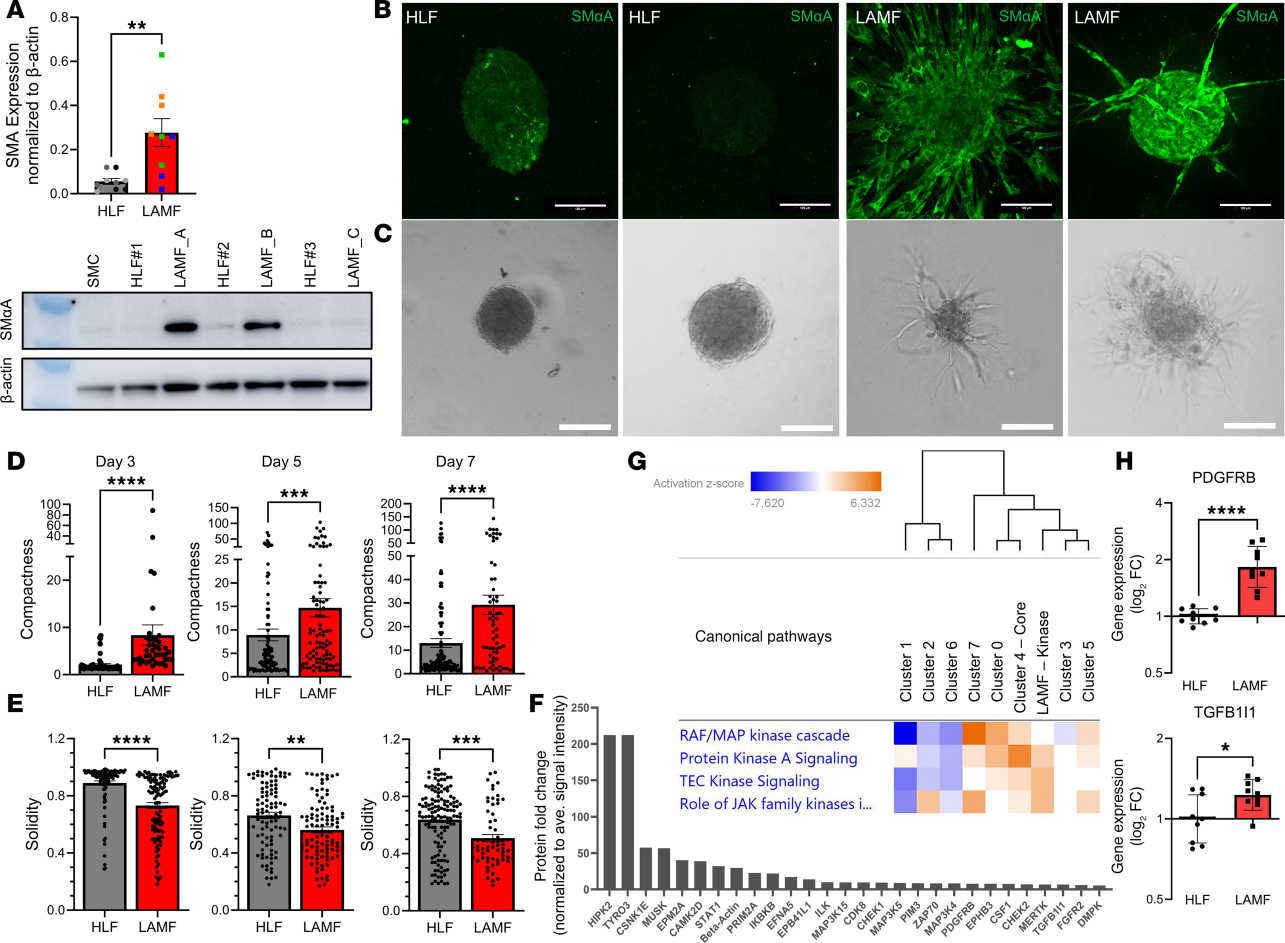
LAMFs represent an activated lung fibroblast phenotype compared with HLFs. (**A**) Relative expression of SMαA comparing HLFs and LAMFs from 3 independent donors with a representative Western blot, with quantification normalized to β-actin. (**B** and **C**) Representative SMαA (green, **B**) and phase contrast (**C**) images of spheroids generated from HLFs and LAMFs; scale bars = 100 μm. (**D** and **E**) Quantification of changes in the compactness (**D**) and solidity (**E**) of spheroids over 7 days comparing LAMFs and HLFs. Each dot indicates a spheroid and a minimum of 11 (range 11–78) spheroids were evaluated. *N* = 3 biological replicates per cell type. (**F**) Kinase array for LAMFs representing expression in LAMFs relative to the average signal intensity across all proteins evaluated. (**G**) Heatmap of canonical pathways comparing the integrated spatial transcriptomics data with the kinase array data showing kinase-related pathway data shown have a cutoff *z*-score > 1 and log_10_*P* value > 1.5; orange is higher pathway activation and blue is pathway inhibition. (**H**) quantitative reverse transcription PCR comparing gene expression of *PDGFRB* and *TGFB1I1* in HLFs and LAMFs. Data represent mean ± SEM. Panels **A** and **H** are analyzed with an unpaired Student’s *t* test and panels **D** and **E** with a Mann-Whitney *U* 2-tailed test with significance represented by *P < 0.05, **P < 0.01, ****P < 0.0001.

We obtained donor-derived LAMFs from UPenn (Supplemental Table 1) and compared these with human lung fibroblasts (HLFs) from donors with no evidence of chronic lung disease (Supplemental Table 1). Despite some donor-to-donor variability, the LAMFs expressed significantly higher amounts of SMαA protein, commonly expressed in contractile smooth muscle cells (Figure 6, A and B). We also evaluated the expression of mTOR and its downstream target P70S6K (Supplemental Figure 4, A and B). Although a significant increase in phosphorylated mTOR was noted in LAMFs, the overall ratio of phosphorylated to unphosphorylated protein was not significantly changed (Supplemental Figure 4A). Despite some donor variability, there was a significant increase in phosphorylated P70S6K and an increase in the ratio of phosphorylated to unphosphorylated protein in the LAMFs, suggesting LAMFs have changed mTOR activity (Supplemental Figure 4B). To verify that P70S6K was phosphorylated downstream of mTOR activity, we showed that rapamycin (Rapa), a specific inhibitor of mTOR, was able to block its phosphorylation (Supplemental Figure 4C). To evaluate functional changes, focusing on matrix invasion, we generated matrix-embedded spheroids. Cell number and media composition were optimized to reproducibly generate spheroids from both HLFs and LAMFs using Aggrewell plates to generate spheroids consistent in size and cell number (Supplemental Figure 5, A and B). After 24 hours the spheroids were embedded in Matrigel, and their growth and invasion into the surrounding matrix were quantified over a 7-day period (Figure 6, B–E). Physical properties of compactness, perimeter, and solidity were measured using an image analysis command pipeline generated in CellProfiler (Broad Institute, detailed in the Supplemental Methods and Supplemental Figure 5, C–E). Each mapped spheroid was checked manually, and manual spheroid edge identification was used where inaccuracies were observed because of low contrast differences between cells and background (Supplemental Figure 5). LAMF spheroids had significantly greater compactness starting from day 3 and sustained through day 7 than HLF spheroids (Figure 6D), indicating a highly irregular shape compared with HLFs. A compact spheroid (circular) would have a value of 1 and irregular objects have a number greater than 1. LAMFs also had significantly decreased solidity than HLF spheroids, again starting at day 3 and sustained through day 7 (Figure 6E), indicating a higher convexity and irregular boundaries as well as being more porous. Interestingly, although trending, there was no significant difference in perimeter (data not shown). The phase contrast images provide representative examples of HLF spheroids and LAMF spheroids after 7 days of 3D culture. Whereas LAMFs were noted to be invading into the matrix, the HLF spheroids typically formed small nodules rather than projections into the ECM (Figure 6, B and C).

As multiple pathways regulating kinases, cell adhesion, and ECM interactions were verified to be transcriptionally upregulated in the LAM-core, we evaluated the expression of a panel of kinases in isolated LAMFs through a kinase array (Figure 6F and Supplemental Data 5). The data shown in Figure 6F highlight the kinases with the highest expression levels in LAMFs; the proteins that had a greater than a 1.25-fold change increase compared with the median protein level of all kinases are featured. The highest expressed protein kinases were HIPK2 and TYRO3 (Figure 6F). However, other notable highly expressed proteins included PDGFRB, MAPK10, MAP4K5, FGFR1, CAMKIID, and TGFB1I1. The heatmap in Figure 6G compares the changes in canonical pathways focused on kinase-related signaling pathways, including the integrated transcriptomics data and kinase array data. Hierarchal clustering showed a close association between the kinase expression in the transcriptomics data for cluster 4 (LAM-core) and the protein kinase array. We evaluated the gene expression levels of *PDGFRB* and *TGFB1I1* in 3 independent donor-derived lines of LAMFs compared with HLFs and verified significantly elevated levels of these genes in LAMFs (Figure 6H). These data support a significant change in fibroblast phenotype in LAM lung tissues.

### Multicellular spheroid models reveal altered fibroblast–endothelial cell interactions in LAM.

**Figure 7:**
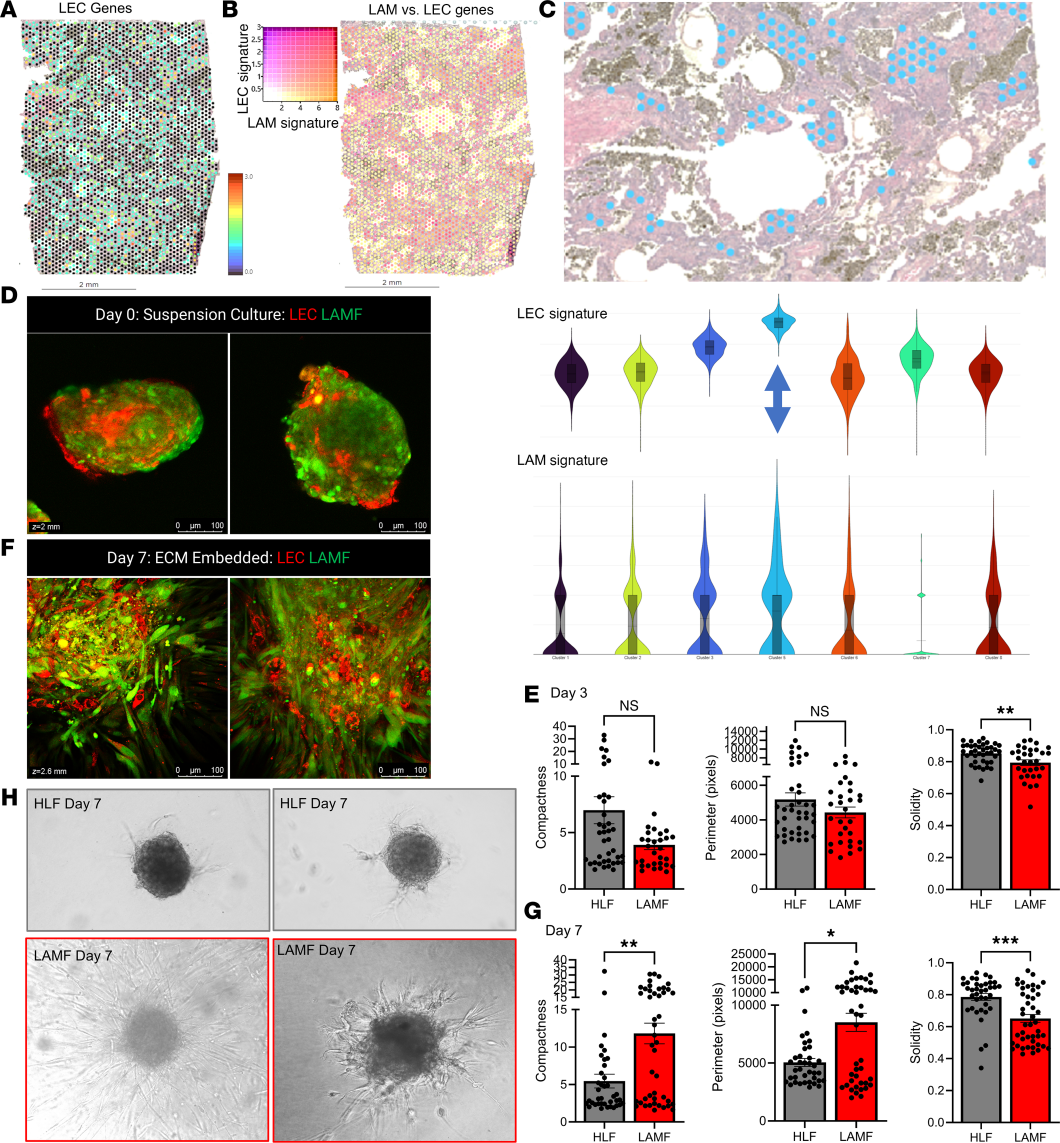
LAMF-LEC organoids have increased invasion into the ECM. (**A**) Spatial heatmap of localization of LEC genes in LAM_D1 tissue (*SOX18*, *PDPN*, *LYVE1*, and *VEGFR3*). Scale bars represent 2 mm. (**B**) Colocalization of core LEC gene signature and LAM-core signature genes in LAM lung tissue LAM_D1. Scale bars represent 2 mm. (**C**) Violin plots showing highest expression of both LEC signature genes and LAM-core signature genes in blue cluster 4, which spatially maps to histological regions of the lung tissue representing LAM nodules. The blue arrow is highlighting the cluster that is represented by the blue dots on the image above (original magnification, ×10). (**D**) Representative immunofluorescence (IF) images of LAMF-LEC spheroids with CellTracker Red–labeled LECs and CellTracker Green–labeled LAMFs 24 hours after seeding in 3D culture conditions. Scale bars represent 100 μm. (**E**) Quantification of changes in the compactness, perimeter, and solidity of the cocultured spheroids over 3 days comparing LAMFs and HLFs. Each dot indicates a spheroid and a minimum of 11 (range 11–78) spheroids were evaluated. (**F**) Representative images of LAMF-LEC spheroids embedded in ECM after 7 days. Scale bars represent 100 μm. (**G**) Quantification of changes in the compactness, perimeter, and solidity of the cocultured spheroids over 7 days comparing LAMFs and HLFs. Each dot indicates a spheroid and a minimum of 11 (range 11–78) spheroids were evaluated. (**H**) Representative phase contrast images of HLFs and LAMFs after 7 days of 3D culture. (Original magnification, ×10.) In all experiments *N* = 3, *n* = 9 experimental repeats. Data shown represent mean ± SEM. Panels **E** and **G** are analyzed using a Mann-Whitney *U* 2-tailed test significance represented by *P < 0.05, **P < 0.01, and ***P < 0.001.

The multiplexed imaging and spatial transcriptomics data highlight close proximity of LECs and activated fibroblasts in the LAM nodules. To begin to understand the complexity of cellular interactions between LAM cells, LAMFs, and LECs in LAM pathogenesis,we next established methodology to reproducibly generate multicellular spheroids comprising LAMFs and LECs (Figure 7). Using *PROX1*, *SOX18*, *LYVE1*, and *PDPN* as a panel of core LEC genes, we spatially mapped LECs onto the lung tissue (Figure 7A). The overlay of these genes with a signature of 5 LAM-core genes (*RAMP1*, *ACTA2*, *PMEL*, *VEGFD*, and *HOXD10*) indicated a close association of both cell types spatially in the LAM tissues (Figure 7B), and transcriptionally these gene signatures were enriched in the LAM-core cluster (Figure 7C, blue). A 3D multicellular model containing both LAMFs/HLFs and LECs was generated to evaluate intercellular interactions in LAM pathogenesis (described in the Supplemental Methods and Supplemental Figure 6, A–C). Commercially available lung microvascular lymphatic endothelial cells (HpMVLECs, referred to herein as LECs) were evaluated for the expression of LEC markers, including expression of PDPN, PROX1, platelet endothelial cell activation molecule (CD31) and vascular endothelial cadherin (CD144). PDPN-expressing LECs comprised 96.5% of the endothelial cells by FACS and IF staining, validating the manufacturer’s description (data not shown). After 24 hours of coculture, the spheroids comprised a green-labeled LAMF core surrounded by red-labeled LECs (Figure 7D and Supplemental Figure 6D). The cellular distribution was verified through staining for SMαA for the LAM cells and PDPN for the LECs (Supplemental Figure 6B). After 3 days of spheroid coculture, there were minimal differences between the HLF and LAMF coculture spheroids (Figure 7E). However, over a 7-day evaluation period, the LAMF-LEC spheroids had significantly higher matrix invasion (Figure 7, F–H) and significantly altered spheroid physical parameters (Figure 7G). The LAMF-LEC spheroids had a significant and substantial increase in spheroid compactness and perimeter, and decrease in solidity, reflecting increased invasion compared with HLF-LEC controls (Figure 7, G and H). Evaluation of live cell images indicated that most of the invading edges of the LAMF-LEC spheroids comprised both cell types (Figure 7F and Supplemental Figure 6E).

### Sorafenib inhibits matrix invasion of LAMF spheroids and LAMF-LEC multicellular spheroids more efficiently than Rapa.

**Figure 8:**
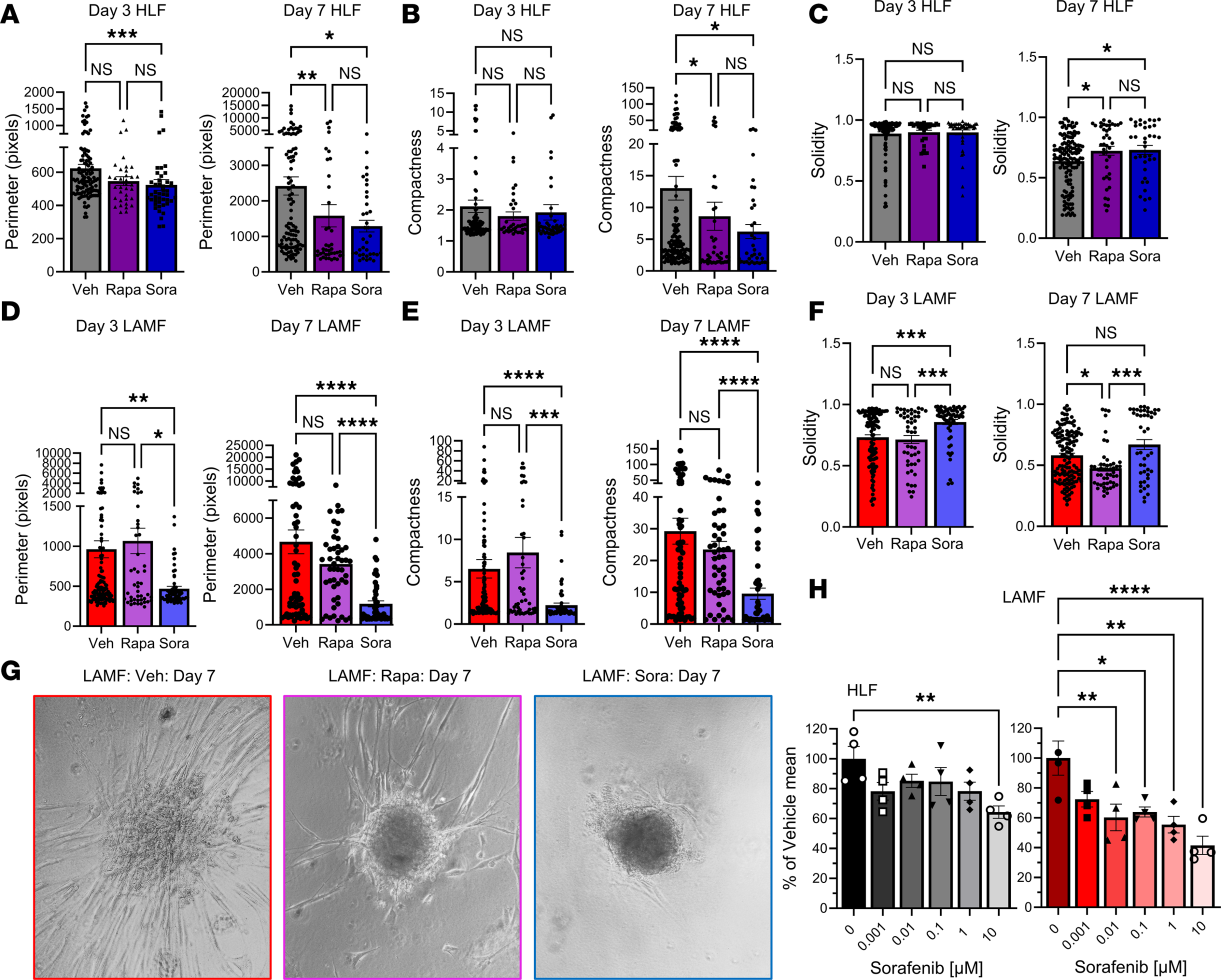
Sorafenib treatment inhibits invasion of LAMF spheroids. (**A**–**F**) Changes in perimeter (**A** and **D**), compactness (**B** and **E**), and solidity (**C** and **F**) comparing day 3 and day 7 for HLF and LAMF spheroids treated with either 20 nM rapamycin (Rapa) or 7 μM sorafenib (Sora), compared with vehicle (Veh). Each dot indicates a spheroid and a minimum of 11 (range 11–78) spheroids were evaluated for each of 3 independent donors (*N* = 3). (**G**) Representative phase contrast images of spheroids at day 7 of treatment for LAMFs comparing Veh with Rapa and Sora treatments. (Original magnification, ×10.) (**H**) Presto Blue viability assays for HLFs and LAMFs in response to increasing doses of Sora; data are expressed as a percentage of the Veh mean from 3 experimental repeats. Data shown represent mean ± SEM; **A**–**F** are analyzed with the Kruskal-Wallis test and post hoc Dunn’s multiple comparisons test. Panel **H** is analyzed with a 1-way ANOVA with post hoc Dunnett’s multiple comparisons tests. Significance is represented by *P < 0.05, **P < 0.01, ***P < 0.001, and ****P < 0.0001, for *N* = 3 independent donor cells.

**Figure 9:**
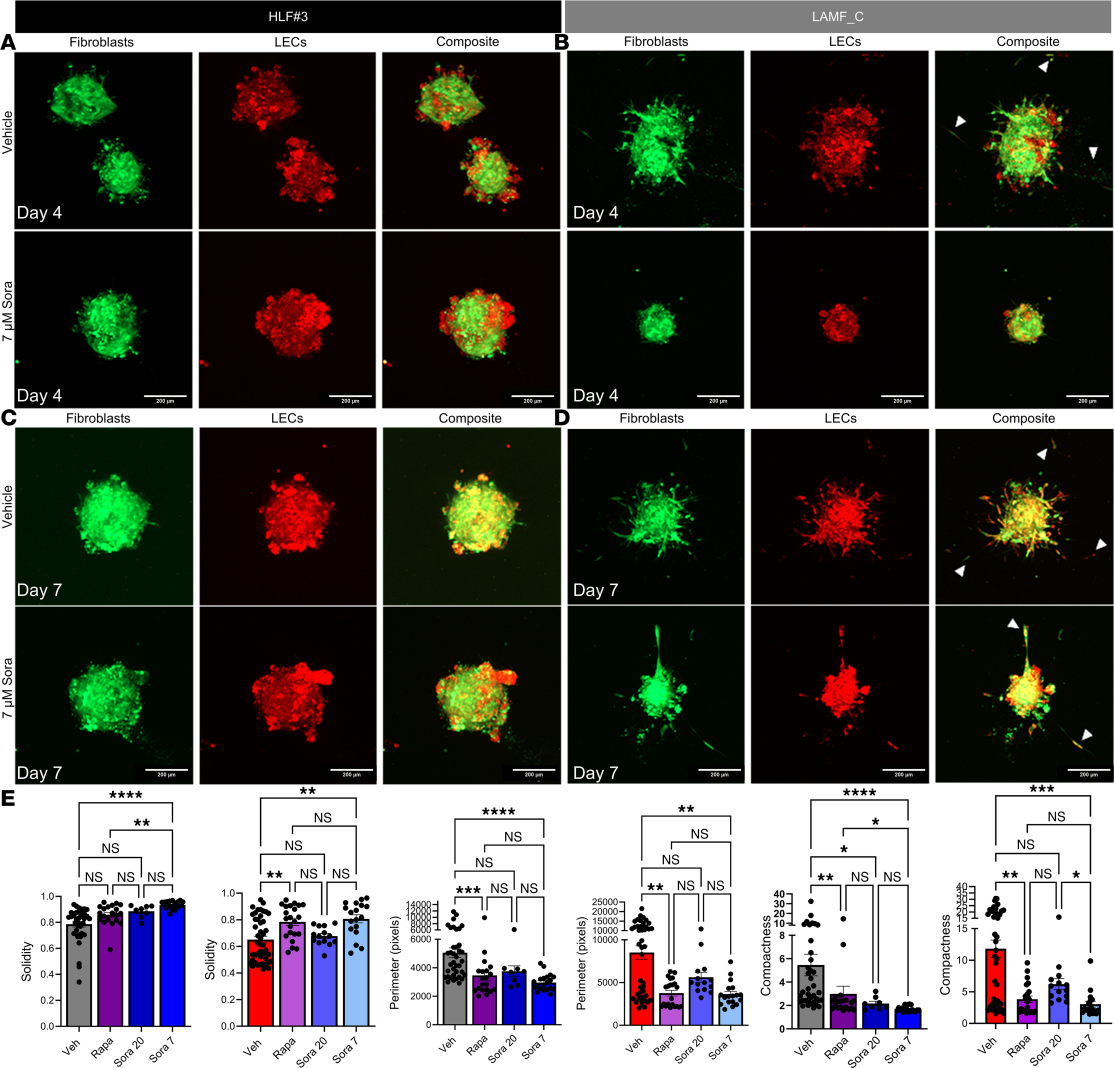
Sora treatment inhibits migration and invasion of LAMF-LEC spheroids. (**A**–**D**) Representative confocal images of spheroids at day 4 (**A** and **B**) and day 7 (**C** and **D**) of treatment with 7 μM Sora or Veh comparing HLFs and LAMFs cocultured with LECs. Fibroblasts are stained with CellTracker Green and LECs with CellTracker Red. Scale bars in all images are 200 μm. (**E**) Changes in solidity, perimeter, and compactness at day 7 for HLF-LEC and LAMF-LEC spheroids treated with either 20 nM Rapa or 20 nM or 7 μM Sora, compared with Veh. Each dot indicates a spheroid and a minimum of 11 (range 11–78) spheroids were evaluated per donor. Data shown represent mean ± SEM. Panel **E** is analyzed with the Kruskal-Wallis test and post hoc Dunn’s multiple comparisons test, and significance is represented by *P < 0.05, **P < 0.01, ***P < 0.001, and ****P < 0.0001 for each of *N* = 3 donors.

LAM cells are known to activate PI3K/AKT and ERK/MAPK signaling (34), in addition to possible induction of angiogenesis through soluble factors such as VEGF-A, PDGF, and VEGF-D targeting VEGFR2-, VEGFR3-, and PDGFRβ-mediated signaling in the LAM microenvironment (16, 48, 52, 53). The data presented in this manuscript highlight the multicellular involvement in LAM pathogenesis and the augmentation of multiple kinase pathways in the LAM-core. We therefore selected an FDA-approved multikinase inhibitor, sorafenib (Sora), to target multiple cell types and signaling pathways to prove the importance of multicellular models and the need for combined signaling pathway targeting in the development of therapeutics for LAM. We selected 7 μM of Sora based on its relevance in in vitro studies and preclinical models (54). Clinically, Sora is administered at a dose of 400 mg twice daily (~13 mg/kg), with in vivo studies employing doses up to 60 mg/kg, corresponding to in vitro concentrations ranging from 3 to 10 μM. We compared Sora with Rapa, an mTOR inhibitor that is the current and only approved therapeutic to slow the progression of LAM (33), in LAMF spheroids and LAMF-LEC multicellular spheroids (Figures 8 and 9). Sora inhibits VEGFR2-, VEGFR3-, and PDGFRβ-mediated signaling targeting the MAPK/ERK pathway to prevent phosphorylation of downstream targets eIF4E and MEK1/2 and ERK1/2 (42–44). To evaluate the effect of Sora on the physical properties of the LAMF and HLF spheroids, we imaged Veh-treated, Rapa-treated, and Sora-treated spheroids daily from day 3 to day 7 of 3D culture. Image analysis revealed an almost complete cessation of invasion in LAMF spheroids at 7 μM Sora, as shown in Figure 8. While a small, but significant, decrease in perimeter (Figure 8A) was noted for Sora-treated HLFs, there were no significant changes in spheroid properties in response to treatment with either Rapa or Sora after 3 days of culture (Figure 8, A–C). In LAMF spheroids, Sora significantly reduced both perimeter (Figure 8D) and compactness (Figure 8E) and increased solidity (Figure 8F), while Rapa had no significant impact on LAMFs at day 3 (Figure 8, D–F). By day 7 Rapa and Sora both significantly and comparably reduced perimeter and compactness and increased solidity in the control HLF spheroids. Interestingly, Rapa had no effect on LAMF perimeter or compactness, while Sora significantly and substantially reduced both the perimeter and compactness to levels closer to those of Veh-treated HLFs (Figure 8, A–F). The phase contrast images in Figure 8G are representative of the changes described above and clearly show the reduction in invasion of LAMFs. Interestingly, a dose-dependent decrease in cell metabolism was noted in Sora-treated LAMFs at doses above 10 nM compared with HLF, which showed changes only at 10 μM, indicating decreased cell viability and increased cytotoxicity specific to LAMFs (Figure 8H). No differences were observed in proliferation between LAMFs and HLFs as shown in Western blots for cell proliferation markers proliferating cell nuclear antigen and cyclin D1 (Supplemental Figure 4D).

To determine whether the presence of LEC affects the activity of Sora on the physical properties of the cocultured LAMF and HLF spheroids, we monitored Veh-, Rapa-, and Sora-treated spheroids daily from day 3 to day 7 of 3D culture (Figure 9, A–D). As shown in the representative images the HLF coculture spheroid morphology did not change considerably in the presence of Sora, with the LEC (red) clustering on the outer edges of the HLF spheroids at day 4 and similar spheroid organization and size observed at day 7 (Figure 9, A–D). On the other hand, LECs cocultured with LAMF spheroids had notable matrix invasion at day 4 (Figure 9, A and B), which was almost completely inhibited in the presence of Sora at both days 4 and 7 of differentiation (Figure 9, A–D). Image analysis revealed an almost complete cessation of invasion in the LEC-LAMF spheroids at 7 μM Sora. Unlike the monocultures, Sora had no significant impact on the cocultured spheroids after 3 days for either HLFs or LAMFs, suggesting some crosstalk between LECs and lung fibroblasts (Supplemental Figure 6, F and G). After 7 days Rapa and Sora treatment significantly reduced perimeter and compactness, with Sora increasing solidity in both HLF and LAMF cocultured spheroids (Figure 9E).

### Intercellular signaling drives changes in cellular phenotype associated with increase of secreted growth factors, VEGF-A and basic FGF, from TSC2-null angiomyolipoma cells.

**Figure 10:**
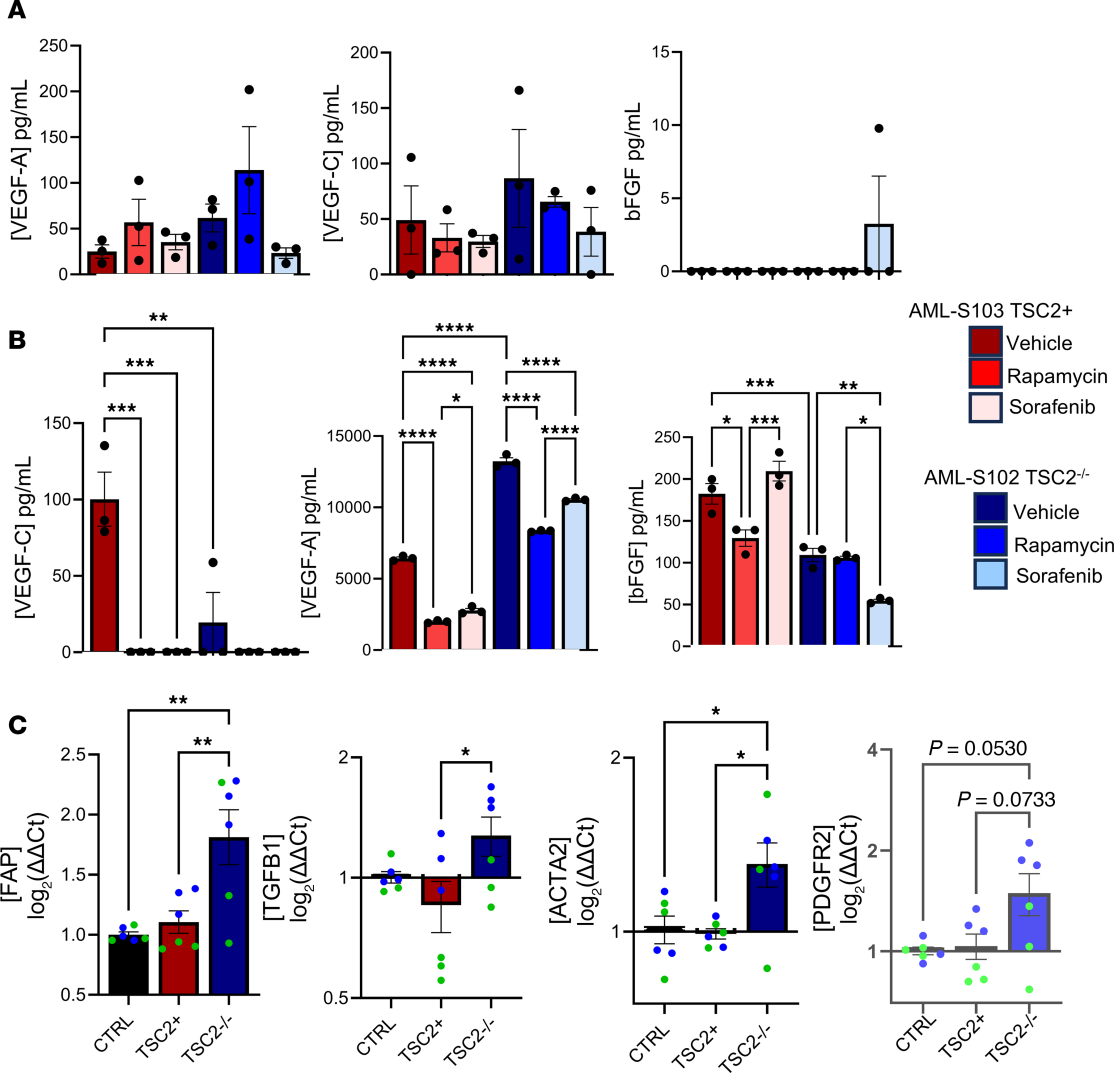
Sora inhibits secretion of pro-angiogenic cytokines from LAMF and *TSC2* AML cells. (**A** and **B**) Secreted VEGF-A, VEGF-C, and bFGF from HLFs (red) or LAMFs (blue) (**A**) and AML S102 (red) and S103 (blue) cells (**B**). (**C**) Gene expression of activated fibroblast markers, *FAP*, *TGFB1*, *ACTA2*, and *PDGFRA*, in HLFs induced by supernatants from either HLFs (black, control) or from AML S103 (red, TSC2^+^) and S102 (blue, TSC2^–/–^) cells. Data shown represent mean ± SEM. All panels are analyzed with a 1-way ANOVA with post hoc Tukey’s multiple comparisons test. Significance is represented by *P < 0.05, **P < 0.01, ***P < 0.001, and ****P < 0.0001 for each of *N* = 3 donors.

**Figure 11:**
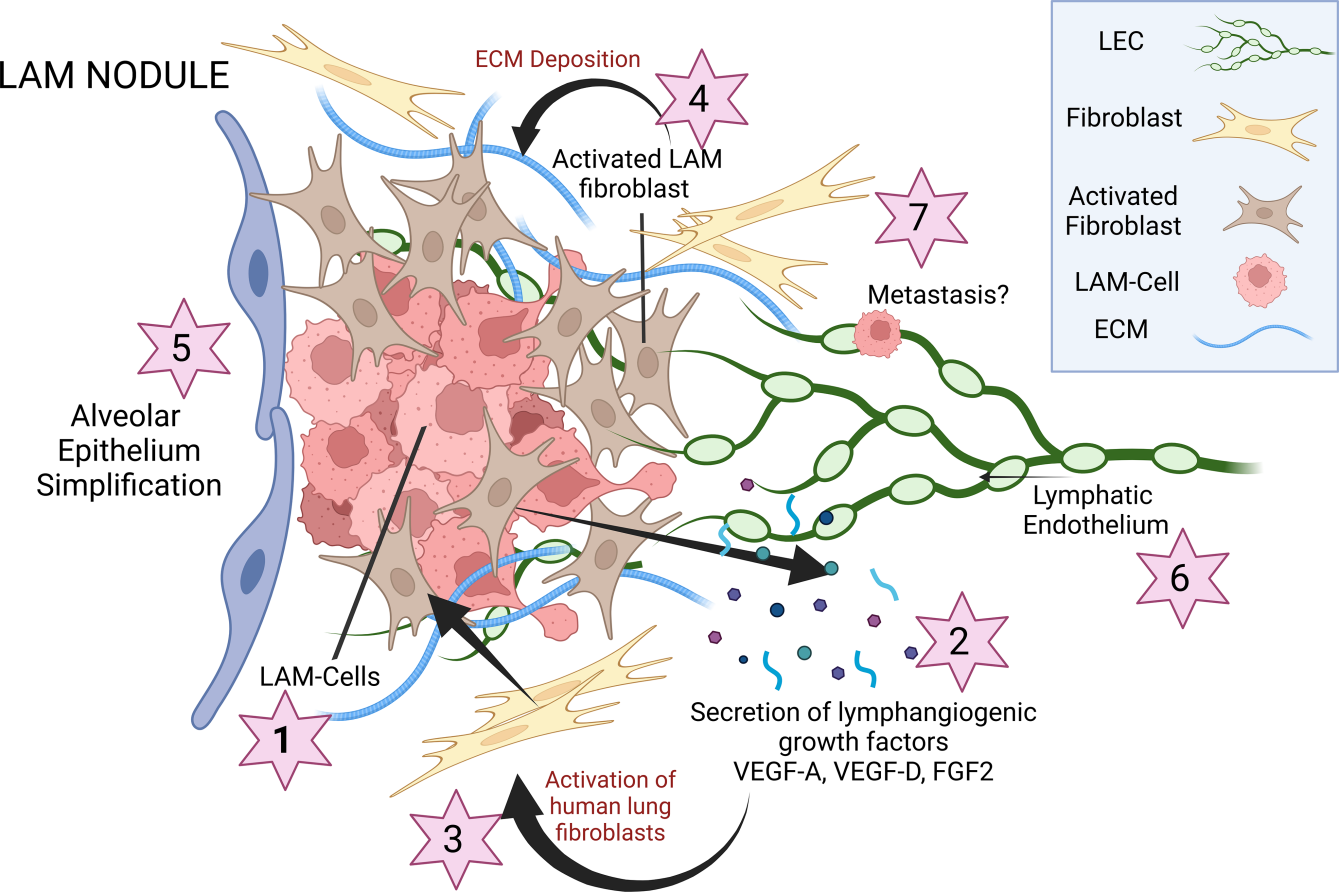
Schematic depicting the proposed cellular interactions leading to LAM pathogenesis. LAM cells (#1) (*TSC2^–/–^*) secrete high levels of VEGF-A, VEGF-D, and FGF2 (#2), which contribute to the activation of resident lung fibroblasts, generating activated myofibroblasts (#3). Activated fibroblasts can lead to dynamic changes in cellular motility and influence the composition of the ECM (#4), creating a unique LAM nodule niche. Activated fibroblasts can also influence alveolar stem cell behavior (#5), and these same secreted factors can recruit LECs (#6) to LAM nodules, which may also provide a pathway for LAM metastasis to other organs (#7).

How LECs are recruited to LAM nodules and whether their tissue invasion is sensitive to changes in LAMFs are currently unknown. Considering that the main alteration and potential priming of both LAMFs and LECs are driven by *TSC2*-null angiomyolipoma (AML) cells, we evaluated changes in secreted growth factors and cytokines from both LAMFs and *TSC2*-null AML cells. *TSC2*-null renal angiomyolipoma cells (AML, S102), and their gene-corrected TSC2 containing control (S103), were shared by Elizabeth Henske, Harvard Medical School (Boston, Massachusetts, USA). *TSC2*-null AML cells have been shown to activate LAMFs, upregulating growth factors, such as FGF7, and subsequently LAMFs can generate soluble factors, potentially influencing alveolar cell phenotype (55). To propose a mechanistic association with the recruitment of LECs to the LAM nodules, we used an angiogenesis multiplexed ELISA panel to determine changes in secreted growth factors and cytokines from HLFs, LAMFs, and S102 and S103 cells in the presence of Veh, Rapa, or Sora (Figure 10). Although trending to increased levels for both VEGF-A and VEGF-C, there were no significant differences in the secreted factors from HLFs and LAMFs in the presence or absence of the inhibitors (Figure 10A). This may be a result of a loss of phenotype with cell passaging in vitro. However, the secretions from the fibroblasts were 30- to 60-fold lower than those observed from the AML cells, indicating that the major changes in secreted factors in the LAM-core are more likely to come from the LAM cells and not the LAMFs. Secretion of VEGF-A, VEGF-C, and basic FGF (bFGF) was significantly different between S102 and S103 and in response to both Rapa and Sora treatment (Figure 10B). Our results show that secretion of VEGF-A, a highly angiogenic growth factor, was significantly augmented in *TSC2*-null AML cells compared with *TSC2*-expressing AML cells, and both Rapa and Sora significantly reduced its secretion in both cell types (Figure 10B). Interestingly, both VEGF-C and bFGF were secreted at significantly higher levels in the *TSC2*-expressing AML cells, with Rapa and Sora completely blocking secretion of VEGF-C in both cell types. A differential effect of Rapa and Sora was observed on the *TSC2*-expressing and -deficient AML cells, with Rapa inhibiting or not changing secretion of bFGF and Sora significantly increasing secretion in *TSC2*-expressing AML cells but significantly inhibiting secretion in *TSC2*-null AML cells. Conditioned media from *TSC2*-null AML cells significantly upregulated gene expression in normal HLFs, leading to activation and phenotypic switching to a myofibroblast (Figure 10C). These data highlight a complex multicellular interaction, responsive to Rapa and multikinase inhibition, and highlight both 1) the necessity of multicellular systems to study LAM pathogenesis and 2) the significant similarity of LAM nodules to the interactions of activated fibroblasts and angiogenesis mechanism in cancer progression (56). Figure 11 highlights the significance of the model and mechanism presented in this manuscript and their potential in driving therapeutic innovation in LAM treatment.

## Discussion

Our study highlights that LAM is a disease driven by complex interactions among multiple cell types, necessitating advanced multicellular models to fully elucidate its pathogenesis and to identify new therapeutic targets. The involvement of multiple signaling pathways further complicates LAM, suggesting that a multifaceted therapeutic approach will be essential to halt disease progression. LAM, as a slowly progressive and metastasizing neoplasm, falls within the family of perivascular epithelioid cell tumors. Although the exact origin of the cells that give rise to LAM nodules remains unidentified, these nodules are known to comprise various cell types, including *TSC2*-null smooth muscle-like cells, HMB-45–positive epithelioid-like cells (LAM cells), LECs, and activated fibroblasts (LAMFs), which closely resemble carcinoma-associated fibroblasts.

Our spatialomics analysis verifies the presence of a set of LAM-core genes, such as *ACTA2*, *TGFBR3*, *HOXA11*, *HOXD11*, *COL14A1*, and *COL3A1*, previously identified through single-cell RNA sequencing as being spatially localized within LAM nodules in patient-derived lung tissues (49). By mapping these LAM-core cells to previously published lung datasets, we found that these cells are most closely associated with myofibroblasts. Myofibroblasts, as activated fibroblasts, play a crucial role in altering the cellular microenvironment by secreting ECM components, growth factors, and cytokines, which often facilitate tumor growth and progression (57–60). We show that normal HLFs respond to factors secreted by *TSC2*-null AML cells (analogous to LAM cells) by upregulating markers characteristic of activated fibroblasts. This finding supports the notion that while bona fide LAM cells may initiate the disease, their interactions with fibroblasts likely drive core pathological changes within the cellular niche, thereby perpetuating disease progression. Indeed, a population of alveolar fibroblasts (AF2s), which also resemble activated fibroblasts, is known to modulate alveolar cell phenotype, leading to alveolar simplification (61). In this study, we used conditioned media from *TSC2*-null AML to evaluate its effect on HLFs. This approach aimed to model the paracrine influence of LAM-like cells on the surrounding lung microenvironment, specifically on fibroblasts, as an early step in disease progression. We did not test the conditioned media on LAMFs, as these cells are already considered to be in a disease-activated state and inherently exhibit many of the features driven by LAM-associated signaling. However, it is possible that LAMFs might respond differently to the conditioned media compared with HLFs, perhaps exacerbating their existing phenotype or altering their secretory profiles further, modeling the chronic nature of LAM. Future studies could involve longer term treatments with conditioned media or coculture systems that include both LAMFs and HLFs to better mimic the disease microenvironment.

In addition, our study highlights the significant interaction between LAM cells and LECs, which migrate into LAM nodules. The changes in the cellular microenvironment, induced by both LAM cells and LAMFs, promote lymphangiogenesis by altering LEC interactions. Notably, we observed a significant increase (Figure 10B) in VEGF-A secretion, a well-known driver of angiogenesis. Augmented VEGF-A expression is strongly linked to ILDs and fibroblast activation, but its role in LAM’s tissue homeostasis and microenvironment regulation is not well understood. Nonetheless, the loss of *TSC2* and subsequent mTOR upregulation, along with increased VEGF-A secretion, has been described previously, supporting our observations in LAM (62). Further investigation into the LAM cell secretome and its impact on HLFs, LECs, and alveolar type 2 cells will likely enhance our mechanistic understanding of the pathological signaling driving LAM, beyond the well-studied mTOR hyperactivation.

Our study underscores that LAM nodules resemble tumor nodules, driven by complex multicellular interactions and multiple signaling pathways. Notably, we demonstrate the utility of mono- and cocultured spheroids to investigate the interaction of LAMFs and LECs, revealing a higher invasive capacity in these cells compared with normal fibroblasts and their cocultures. This increased invasiveness is associated with significant upregulation of key signaling molecules, including VEGF-A, VEGFR2, and bFGF, suggesting a pro-tumorigenic mechanism within the LAM nodule microenvironment. Tumors with significant heterogeneity, including LAM nodules, often express PDGFRB and have been shown to attract LECs through the PDGF/PDGFRB axis (63), reinforcing the pathway for LAM cell–induced lymphangiogenesis and potential metastasis through lymphatic circulation (16, 17). Additionally, the pro-neoplastic role of PDGF is further supported by its contribution to collagen formation, which may increase remodeling of the LAM microenvironment (64).

Furthermore, our study highlights the potential of multikinase inhibitors, such as Sora, in targeting these signaling pathways. Rapa, the primary drug of choice according to treatment guidelines, is currently only available for patients with reduced lung function in progressed LAM, and while most of these patients do respond well, resulting in stabilization of lung function, it is not effective for all (35, 65–68). Rapa is also known to be primarily cytostatic and not cytotoxic, limiting its capacity for inhibition of tumorous growth and highlighting the need to identify new therapeutic targets for the treatment of LAM (66–69). Sora is an FDA-approved multikinase inhibitor (41–43) that is known to inhibit RAF/MEK/ERK signaling, leading to increased tumor cell apoptosis, decreased microvessel density, and reduced metastatic shedding of tumor cells (41–44) and inhibiting the phosphorylation of eIF4E, which is typically upregulated in LAM, downstream of the upregulated mTOR pathway (3, 43–45). In our model, Sora demonstrated superior efficacy compared with Rapa in suppressing LAMF invasion into the surrounding ECM, indicating its potential as a therapeutic option in advanced LAM by simultaneously targeting LAMFs and LECs. Previous studies have shown that Sora and other multikinase inhibitors can effectively impede tumor progression and reduce the likelihood of recurrence and metastasis (41–43). Our findings suggest that a combination of low-dose Sora with mTOR inhibitors, or even low-dose Sora monotherapy, may represent a promising therapeutic strategy for managing end-stage LAM, particularly in patients with high VEGF-D levels who are unresponsive to Rapa. VEGF-D, currently utilized as a diagnostic biomarker for LAM with a serum cutoff value of approximately 800–1,000 pg/mL, has shown a significant correlation with disease severity (40, 69–72). Additionally, lower diffusion capacity of the lung for carbon monoxide (DLCO) scores, which serve as functional markers of interstitial involvement and reflect tissue-level changes, such as fibroblast activation and lymphatic disruption, have been associated with higher mortality in ILD (73–75). To enhance LAM management, refining VEGF-D cutoff levels through clinical studies or developing a comprehensive clinical scoring system that incorporates DLCO, FEV_1_, VEGF-D levels, and the assessment of lymphatic abnormalities could improve patient stratification and therapy effectiveness. Such an approach could be instrumental in assessing the efficacy of therapies like multikinase inhibitors. Ongoing clinical studies on nintedanib, a multikinase inhibitor targeting FGFR, PDGFR, VEGFR, and TGF pathways, have shown potential in stabilizing fibrotic lung diseases like idiopathic pulmonary fibrosis, but its effectiveness in LAM remains under investigation (76, 77). This highlights the importance of exploring a broader spectrum of kinase inhibitors for effectively halting LAM progress under Rapa, suggesting a more potent action beyond mere cytostatic activity.

Looking ahead, focused research on the LAM cell secretome is crucial to uncover the mechanisms driving LAM cell detachment and intravasation and their impact on the lymphatic endothelial barrier. Understanding the interactions between LECs, fibroblasts, and LAM cells will provide critical insights into how LAM cells invade the lymphatic system and spread to other organs. This comprehensive understanding of LAM pathogenesis will be pivotal in developing innovative therapeutic strategies. In conclusion, our study reinforces the classification of LAM as a slow-growing neoplastic disease with a complex microenvironment driven by multiple cell types and signaling pathways. These findings not only open new avenues for therapeutic intervention but also underscore the need for continued research into the pathogenesis of LAM, with the ultimate goal of developing more effective treatments for this challenging disease.

## Methods

Detailed methodology is found in the Supplemental Methods, which can also be accessed at doi: 10.6084/m9.figshare.26880469.

### Sex as a biological variable.

Our study focused on female patients because LAM is a disease that almost exclusively affects women. We believe the findings are highly relevant to women because of the nature of the disease. Since LAM is extremely rare in men, it is uncertain whether these results would apply to the male population.

### Study design.

The objective of this study was to create a coculture 3D model to evaluate cellular interactions between LAMFs and LECs in the pathogenesis of LAM. Our work included multiplexed immunohistochemistry and spatialomics analysis of LAM donor lung tissues, characterization of LAMFs compared with HLFs, quantitative evaluation of LAMFs and HLFs and cocultures with LECs in 3D spheroid cultures, and comparison of the effectiveness of the current first-line therapeutic, Rapa, and a multikinase inhibitor on LAMF invasion. Cells and tissue samples were selected based on the diagnosis for the donor lung tissues, having LAM or nonsmokers with no prior history of chronic lung disease. For histological experiments, 3 LAM lung tissues were used. For spatialomics analysis 2 LAM lung tissues were used. For all other experiments a minimum of 3 biological replicates from diseased (LAM) and controls (HLF) were used. For all 3D culture analysis a minimum of 12 wells of spheroids were used per condition. Outliers were recorded and included.

### Invasion assay and image analysis.

Fibroblast-LEC spheroids were created through the sequential addition of primary human pulmonary lymphatic microvascular endothelial cells (HpMVLECs, Angio-Proteomie) to the fibroblast spheroids. Cells were plated in equal ratios with 450,000 LECs being added to spheroids comprising 450,000 fibroblasts in each Aggrewell well. This results in each spheroid containing approximately 1,500 cells of each type. To monitor the growth and invasion, daily phase contrast images were collected over 7 days. To evaluate morphology and phenotype of spheroids/organoids, an automated pipeline was developed using CellProfiler (78).The pipeline was designed to enhance specific aspects of the phenotype and accurately identify both primary and secondary objects, including spheroid cores and spheroid sprouts (see Supplemental Figure 3). The modified pipeline allowed for a more precise quantification of various features, including size and shape, and facilitated the analysis of the spheroid/organoid behavior under different experimental conditions. Image analysis parameters were 1) Compactness: A filled circle will have a compactness of 1, with irregular objects or objects with holes having a value greater than 1. 2) Perimeter: Total length of the perimeter of the objects image. 3) Solidity: Equals 1 for a solid object or <1 for an object with holes or possessing a convex/irregular boundary. 4) Form factor: Calculated as (4 × π × area)/perimeter^2^. Equals 1 for a perfectly circular object, >1 for an irregular object.

### Visium 10x Genomics spatialomics profiling.

Spatialomics profiling was performed in collaboration with the Iowa NeuroBank Core in the Iowa Neuroscience Institute (INI) and the Genomics Division in the Iowa Institute of Human Genetics (IIHG) and followed manufacturer’s instructions (Doc. CG000409, Rev. A; Visium, 10x Genomics). Briefly, 2 independent regions from 1 LAM lung donor were used to create 10 μm sections for quality control in the Comparative Pathology Laboratory in the Department of Pathology, University of Iowa. Sections were H&E-stained and regions of interest selected based on the presence of LAM nodules and cysts. RNA quality was determined by extracting RNA with the QIAGEN RNeasy FFPE kit, measuring RNA concentration using a Qubit fluorometer (Thermo Fisher Scientific), and evaluating the samples using a 4200 Tapestation (Agilent) in the Genomics Division of the IIHG. For sequencing, 10 μM FFPE sections were rehydrated, then adhered to the Visium Spatial Gene Expression Slide (PN-2000233, 10x Genomics) in a 42°C water bath. Samples were dried in a desiccation chamber and deparaffinized using QIAGEN Deparaffinization Solution for 2 hours at 60°C. After H&E staining, bright-field tile scans of each complete section area were captured and stitched together using an Echo Revolution microscope with a 20× objective (software version 1.0.26.2). De-cross-linking was performed according to manufacturer’s protocol (Doc. CG000407, Rev. D) and immediately hybridized to the Visium Human Transcriptome Probe Kit V1.0 (10x Genomics), which contained 19,144 genes targeted by 19,902 probes. After probe extension, sequencing library construction was performed using unique sample indices using the Dual Index Kit TS, Set A (PN-1000251, 10x Genomics), for Illumina-compatible sequencing. Paired-end sequencing (2 × 100) was performed on the Illumina NovaSeq 4000.

### Statistics.

All data are presented as mean ± SEM. For determining statistical significance among 2 groups, a Student’s unpaired *t* test (2-tailed) was used. When multiple groups were evaluated, either a 1-way ANOVA with post hoc Dunnett’s or Tukey’s multiple comparisons test or a Kruskal-Wallis with post hoc Dunn’s multiple comparisons test was utilized for pairwise comparisons between the groups. Other statistical tests are indicated in the figure legends. Statistical significance was considered at the 5% level, with a value of *P* < 0.05 being considered statistically significant. Unless otherwise stated, the data were collected from a minimum of 3 independent donor (*N*) and 3 independent experimental replicates (*n*).

### Study approval.

Deidentified LAM lung samples were obtained from living donors at the time of lung transplantation through the National Disease Research Institute (NDRI; Philadelphia, Pennsylvania, USA) protocol number RKRV1. Informed consent was obtained by NDRI before acceptance of tissue donation for research. Identifying information was removed before sample use in accordance with institutional and NIH protocols. Human lung tissue from patients with no prior history of chronic lung disease was obtained from the International Institute for the Advancement of Medicine, the Cystic Fibrosis Foundation Tissue Procurement and Cell Culture Core at University of North Carolina at Chapel Hill, or the University of Iowa in collaboration with Kalpaj Parekh, with approval from the Institutional Review Board of the University of Southern California (Protocol number: HS-18-00273).

### Data availability.

All data values within this study are present in the paper or available in the supplement (corresponding Supporting Data Values XLS file or doi: 10.6084/m9.figshare.26880469). The spatialomics datasets, including raw sequencing data and processed files, have been deposited at NCBI GEO under the series reference GSE234885 (datasets GSM7476184 and GSM7476185). This paper includes original code, which is available in the GitHub repository (https://github.com/gautam-lk/RyanLab_LAM; commit ID eda3311). In addition, all raw data are available at doi: 10.6084/m9.figshare.23464976.

## Author contributions

SKG and ALR conceptualized the study. SKG, ECL, LKG, BAC, SM, NCH, JCN, and ALR carried out the investigation. SKG, ECL, LKG, SM, JCN, and ALR analyzed data. SKG and ALR wrote the original draft of the manuscript, which was reviewed and edited by SKG, ECL, LKG, VPK, and ALR and given final approval by all authors. SKG, BZ, and ALR provided resources and supervision. SKG and ALR are the guarantors of this work and, as such, had full access to all the data in the study and take responsibility for the integrity of the data and accuracy of data analysis.

## Supplementary Material

Supplemental data

Supplemental data set 1

Supplemental data set 2

Supplemental data set 3

Supplemental data set 4

Supplemental data set 5

Unedited blot and gel images

Supporting data values

## Version 1 02/04/2025

In-Press Preview

## Version 2 03/24/2025

Electronic publication
