# Supplementary material for: Targeting fibroblast–endothelial cell interactions in LAM pathogenesis using 3D spheroid models and spatial transcriptomics
Source: JCI Insight. 2025 Feb 4;10(6):e187899. doi: 10.1172/jci.insight.187899 (PMC11949067; doi:10.1172/jci.insight.187899)

Figure 4

Full aSMA and Bactin blots

Images on this slide correspond to blots in Figure 4 Panel A

Representative images _ blot #2

Red = portion of the blot in the figure

α-SMA (42 kDa)

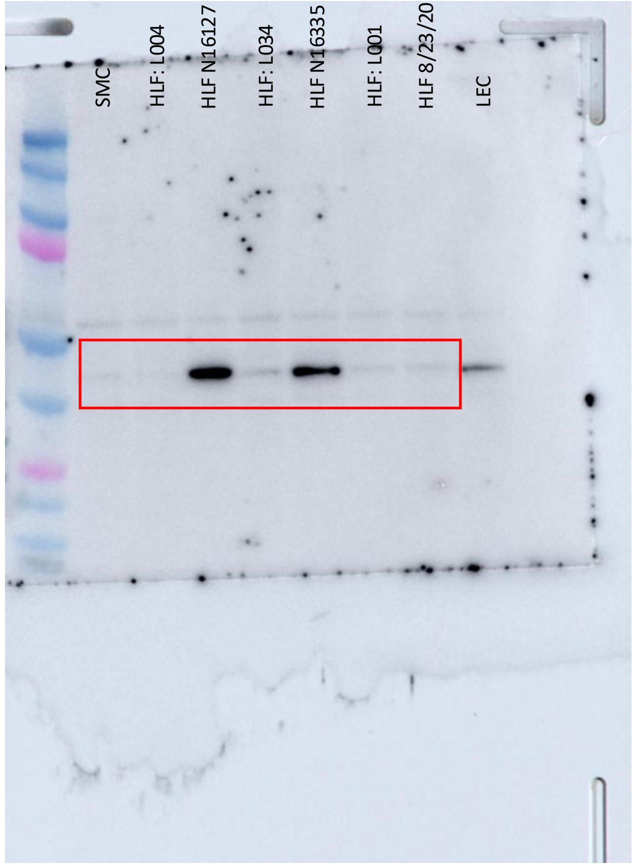

42 kDa
Mouse ab

B-actin (46 kDa)

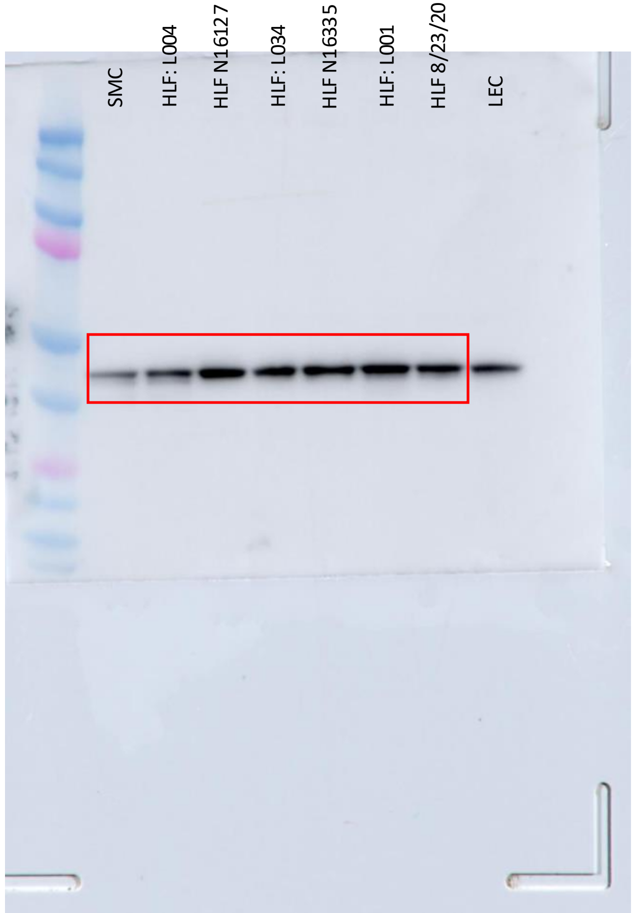

46 kDa
Rabbit ab

Images on this slide correspond to blots in Figure 4 Panel A

a-SMA Images used for analysis

42 kDa
Mouse ab

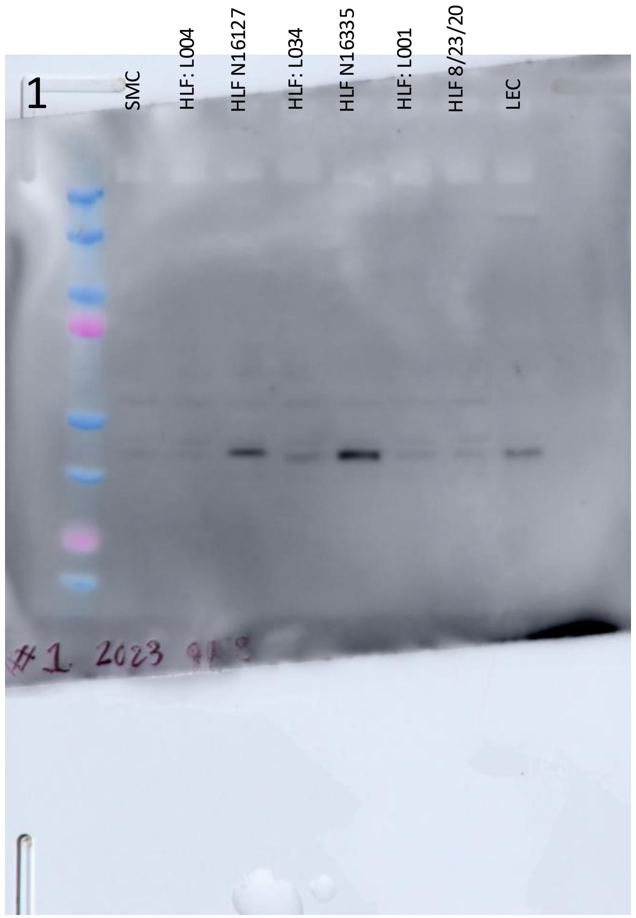

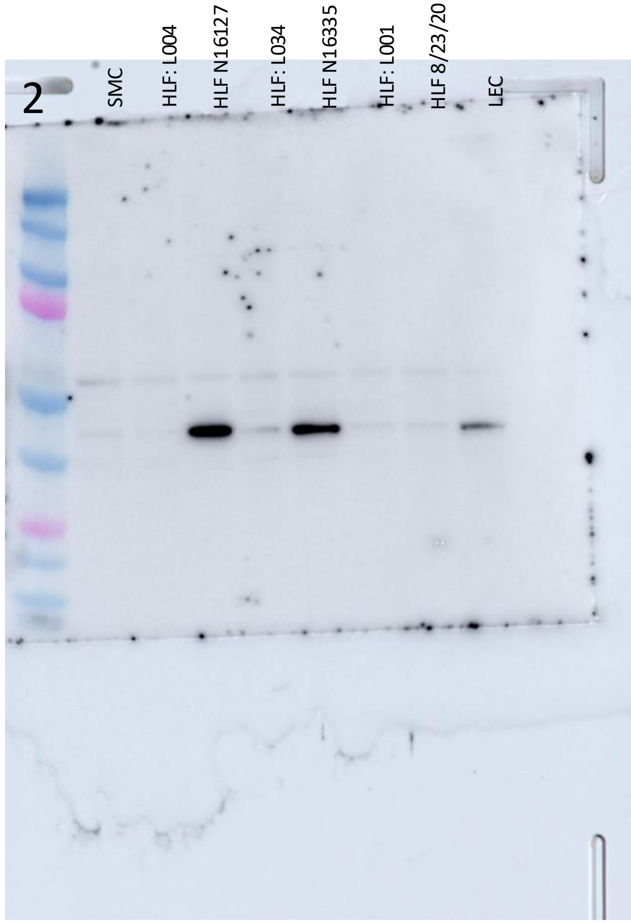

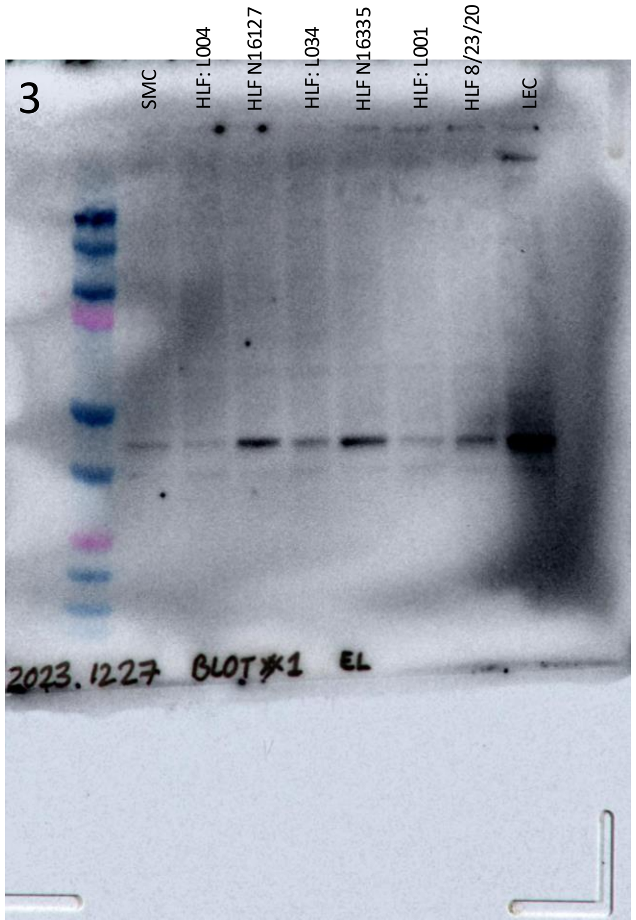

Images on this slide correspond to blots in Figure 4 Panel A

B-actin Images used for analysis

46 kDa
Rabbit ab

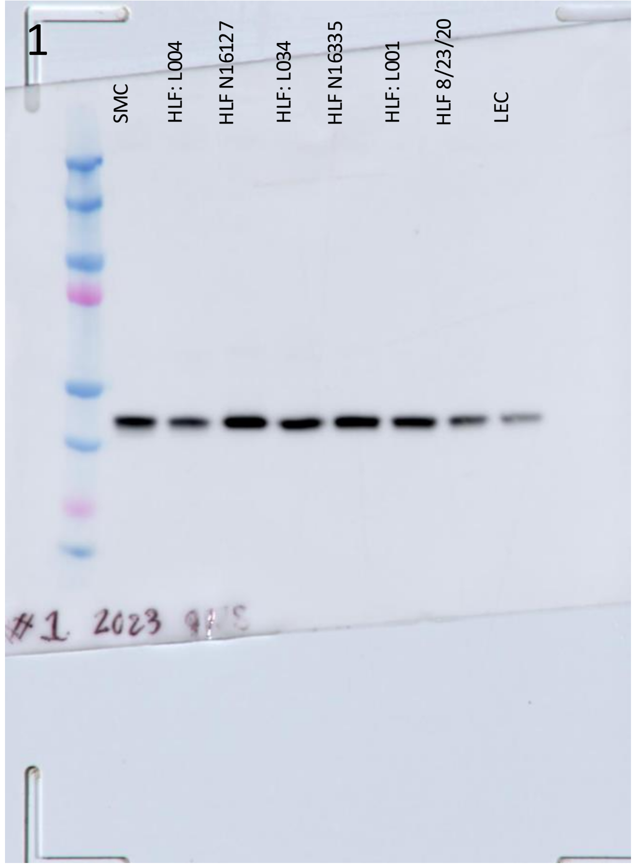

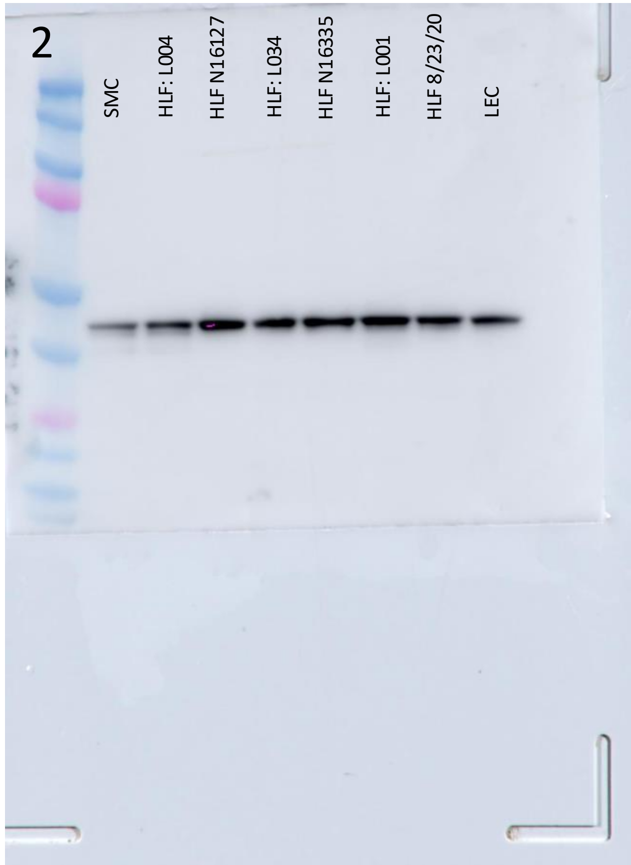

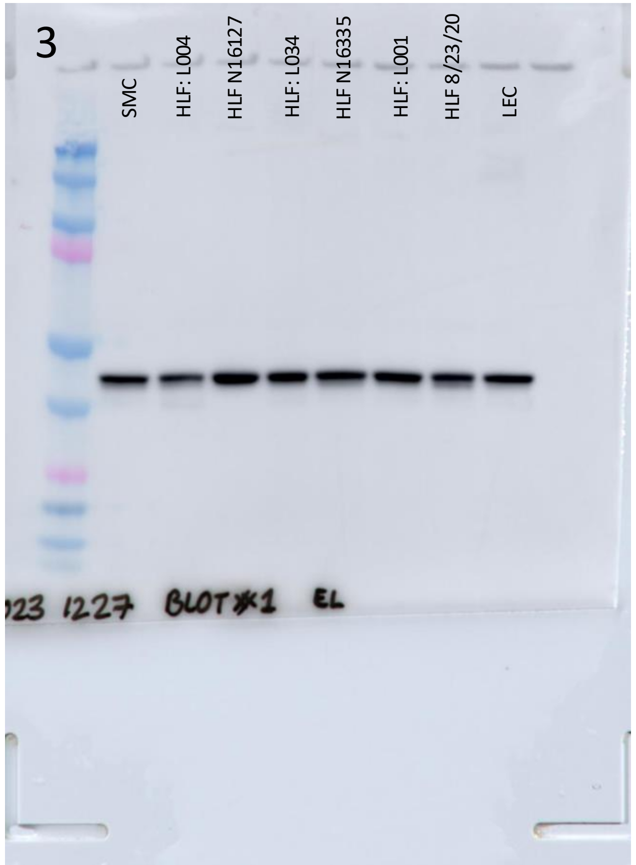

Supplement: Unedited blot and gel images [file jciinsight-10-187899-s152.pdf]
